# Exploring deep learning capabilities for surge predictions in coastal areas

**DOI:** 10.1038/s41598-021-96674-0

**Published:** 2021-08-26

**Authors:** Timothy Tiggeloven, Anaïs Couasnon, Chiem van Straaten, Sanne Muis, Philip J. Ward

**Affiliations:** 1 Institute for Environmental Studies (IVM), Vrije Universiteit Amsterdam, Amsterdam, The Netherlands; 2 KNMI, Utrechtseweg 297, 3731 GA De Bilt, The Netherlands; 3 Deltares, Delft, The Netherlands

**Keywords:** Climate sciences, Environmental sciences, Natural hazards, Ocean sciences

## Abstract

To improve coastal adaptation and management, it is critical to better understand and predict the characteristics of sea levels. Here, we explore the capabilities of artificial intelligence, from four deep learning methods to predict the surge component of sea-level variability based on local atmospheric conditions. We use an Artificial Neural Networks, Convolutional Neural Network, Long Short-Term Memory layer (LSTM) and a combination of the latter two (ConvLSTM), to construct ensembles of Neural Network (NN) models at 736 tide stations globally. The NN models show similar patterns of performance, with much higher skill in the mid-latitudes. Using our global model settings, the LSTM generally outperforms the other NN models. Furthermore, for 15 stations we assess the influence of adding complexity more predictor variables. This generally improves model performance but leads to substantial increases in computation time. The improvement in performance remains insufficient to fully capture observed dynamics in some regions. For example, in the tropics only modelling surges is insufficient to capture intra-annual sea level variability. While we focus on minimising mean absolute error for the full time series, the NN models presented here could be adapted for use in forecasting extreme sea levels or emergency response.

## Introduction

In order to improve coastal adaptation, it is critical to better understand and predict the characteristics of sea levels, such as their temporal variation and magnitude over long time periods, typically multiple decades^1,2^. Coastal sea level variability results from a combination of multiple processes, including mean sea level variations, tides, waves and storm surges resulting from the passing of low pressure systems and strong winds^3,4^. We focus here on the non-tidal residual, also referred to as surge or surge residual, i.e. the water level after removal of the tide and mean sea level. At hourly time scales or shorter, temporal variation in surge residuals depends on the direction and variations in wind and pressure gradients, as well as local topographic characteristics such as the bathymetry and complexity of the coastline^5–7^. Surge levels superimposed on high tides can exceed land thresholds and contribute to nuisance flooding or extreme impacts when caused by tropical or extratropical cyclones. At the global scale, studies have used hydrodynamic modelling^8^ or data-driven approaches to reconstruct surge time series^9–12^. The advantage of hydrodynamic models is that with adequate model resolution and meteorological forcing, they can resolve physical coastal processes and their interactions. They are also valuable for understanding epistemic uncertainties and the relative contributions of different oceanographic and coastal processes in total water levels. However, these models are computationally demanding and take a long time to set up^13–15^. This limits their ability to be used in the simulation of large ensembles of events^16,17^.

To circumvent these limitations, studies have applied data-driven models to predict surge at gauged locations, such as empirical relationships^18^, unsupervised learning algorithms^10,19^ or approaches using artificial intelligence like deep learning^11,20–22^, and found comparable or even better performance compared to hydrodynamic models. For example, Tadesse et al.^10^ found the daily maximum surge levels from their data-driven model to outperform the global hydrodynamic model from Muis et al.^23^. They applied random forests and linear regression, selecting as predictors a range of atmospheric (wind speed, mean sea level pressure, precipitation) and oceanographic (sea surface temperature) variables. These predictor variables were selected with various lag-times in a 10 × 10 degree box around the location of interest from remotely sensed satellite products and climate reanalysis datasets. Among deep learning approaches, Artificial Neural Networks (ANN) have been popular Neural Network (NN) models for operational surge level forecasting^22,24,25^ or the modelling of stochastic storm surge events^26,27^. Although limitations exist when applying NN models, such as capturing long term processes and predicting at ungauged locations, applying such methods can lead to similar or better results than local hydrodynamic models. At the global scale, Bruneau et al.^11^ were, to our knowledge, the first to use ANN models to predict hourly non-tidal residual levels at tide stations. They used as predictor variables wind, mean sea level pressure, accumulated precipitation, and wave height from the climate reanalysis dataset ERA5^28^. The spatial extent of the predictor variables around each location considered was four times smaller (i.e. 5 × 5 degree box) than used in Tadesse et al^10^. Due to its refined horizontal resolution, this dataset can better resolve characteristics of climate extremes, such as tropical cyclones (track, intensity, maximum wind speeds) than its predecessor ERA-Interim reanalysis^29^ even though some improvements are still needed, for example to properly capture their outer size^30^.

Notwithstanding the differences between the models applied in Tadesse et al.^10^ and Bruneau et al.^11^, the role of the number of predictor variables considered and the spatial extent around each location in the model’s performance and ability to learn remains unclear. Next to ANNs, other NN types could be useful for global scale surge level prediction^31,32^. For example, Convolutional Neural Networks (CNN) can process patterns in spatio-temporal climate data and identify weather features to be used for forecasts^33,34^. Recurrent neural networks using Long Short-Term Memory (LSTM) layers have been used in hydrology to capture long-term temporal dependencies, necessary to capture the state of a river basin^35,36^.

In this paper, we explore the capability of different deep learning approaches to predict surge levels at the global scale. To do so, we predict hourly surge using four types of NN and evaluate their predictive skill. We train, validate and test a CNN, LSTM and combined CNN-LSTM (ConvLSTM) model to capture spatial, temporal, and spatio-temporal dependencies for surge level observations from 736 tide stations. We benchmark our NN models with a simple probabilistic reference model based on climatology. Next, for 15 selected locations with diverse surge characteristics, we examine the NN skill gained from increasing the spatial extent considered around each location and the number of different variables used as predictors. Additionally, we show the capability of the four NN types to gain skill when adding complexity to their respective network architecture.

## Methods

We predict hourly surge at tide stations from the Global Extreme Sea-Level Analysis Version 2 database (GESLA-2)^37^ using four different deep learning models following the main steps described in this section. In brief, we extract the predictand from the GESLA tide stations and predictor variables from the atmospheric reanalysis ERA5 from ECMWF^28^. For each station, we construct and run four NN model types and compare their performance with observed surge levels and with a simple probabilistic model as benchmark. Finally, for fifteen stations, we analyse the influence of the number of predictor variables, the spatial extent considered around each location (from hereon called spatial footprint), and the architecture of the NN on its performance.

### Data preparation

#### Predictand variable: surge time series

We use total water levels from the GESLA-2 dataset, a quasi-global dataset of sea levels at a high temporal frequency (15 min or one hour) for 1276 stations. Each time series is resampled to an hourly frequency for consistency. The dataset has already been thoroughly controlled to flag any potential erroneous signal, for example for tsunamis and was thus not further inspected^37^. We do not interpolate between periods with no data. The following steps are applied to extract the surge time series and are illustrated in Figure S1. Since this study focuses on surge prediction, we remove inter-annual mean sea-level variability by subtracting the annual moving average (365 days) (Fig. S1a). We decompose the de-trended sea-level time series into the tide and non-tidal residual by applying a harmonic analysis using the UTide (Unified Tidal Analysis and Prediction Functions) Matlab package^38–41^ (Fig. S1b). UTide uses an automated decision tree to select the most important constituents from 146 tidal constituents and performs a nodal correction for time series longer than 2 years. A comparison with tidal predictions from NOAA at three stations with contrasting tidal environments indicates differences are mainly within +/− 5 cm (Figure S2). This is in line with the errors typically found from extracting the tide from observed water level series^42,43^. A drawback of a harmonic analysis is that the residual time series often contain a remaining tidal signals due to small phase shifts in the predicted tide^41,44,45^. At a daily time scale, using the skew surge overcomes this problem^46^ and at an hourly time scale, low-pass filtering methods, such as the recursive Chebyshev Type II filter, have been recommended to fully remove this component^47,48^. Here, we select a simple filter and apply a 12-h moving average to limit the influence of spurious peaks in the predicted tide (Fig. S1c). This final time series is considered as the predictand variable, i.e. the surge. We investigate to which extent this filter could impact sea-level extremes at three stations, see Figure S3. The amount of under of overestimation is highly dependent on the relative contribution of the tides and surge in sea-level extremes. A clear advantage however from using this filtering approach is that extreme value analysis becomes more robust to errors in timing.

**Figure 1:**
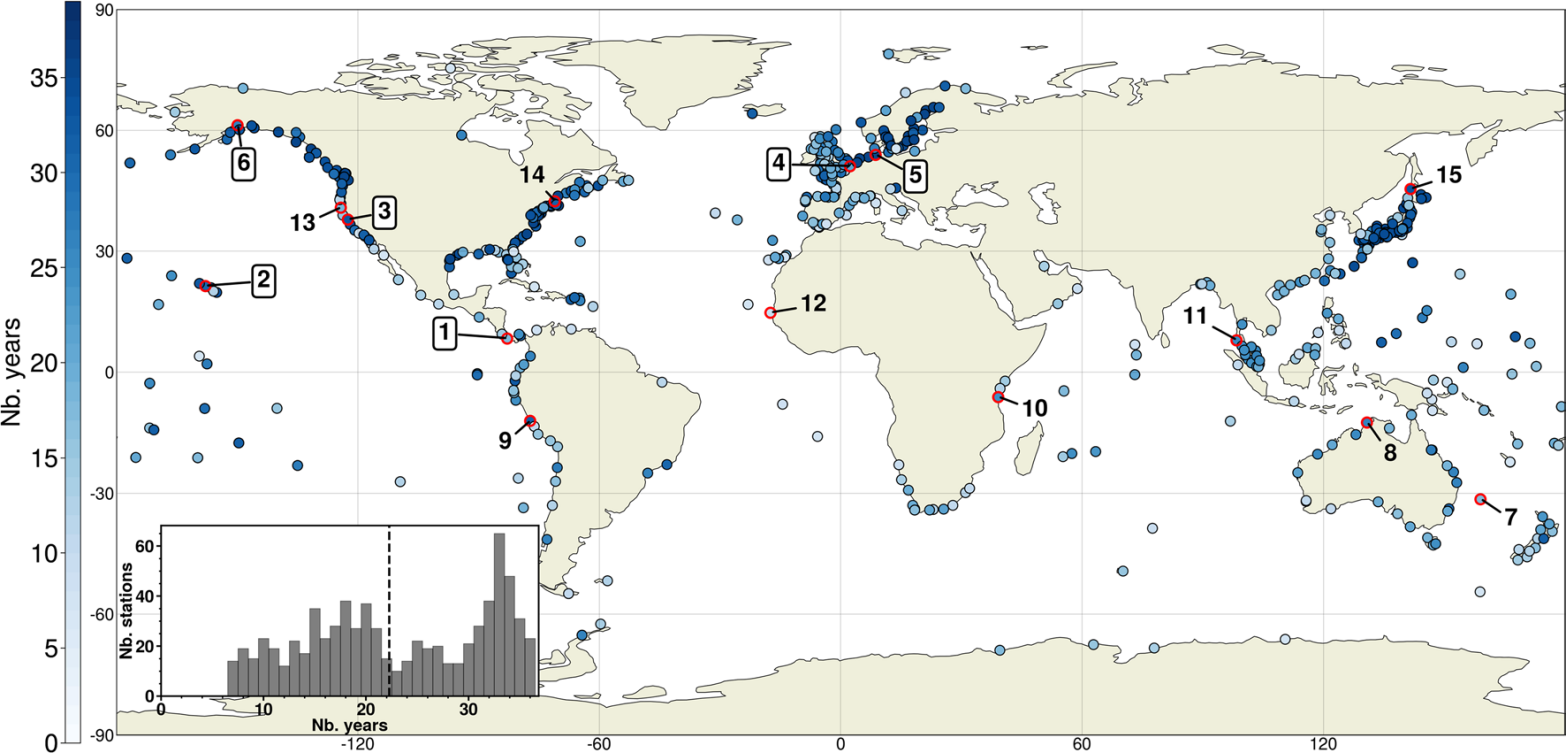
Tide stations considered in this study. Inset figure presents the histogram of the length of the data records with the median value shown by a dashed line. The set of 15 stations selected for further analysis is circled in red: 1-Puerto Armuelles (Panama), 2-Honolulu (Hawaii), 3-San Francisco (USA), 4-Dunkerque (France), 5-Cuxhaven (Germany), 6-Anchorage (USA), 7- Lord Howe (Australia), 8- Darwin (Australia), 9-Callao (Peru), 10-Zanzibar (Tanzania), 11- Ko Taphao (Thailand), 12- Dakar (Senegal), 13- Humboldt Bay (USA), 14- Boston (USA), 15- Wakkanai (Japan). Results for the boxed numbers (1–6) are shown in the main study and the rest (7–15) in supplementary.

In order to train, validate and test the NN models, we select all stations that have at least seven years of data between 1979 until 2019. Bruneau et al.^11^ found that a minimum of six years of training data is needed to obtain stable NN skill. We select an additional year of data that is neither used in the training nor the validation, to test model performance. Therefore, the seven years of data should at least consist of one consecutive year without gaps for testing, and at least 6 years of consecutive sequences (10 days) without missing data (more details in “Neural network models and skill metrics” section) for training and validation. This leads to a set of 736 stations (Fig. 1). To get more insight into regional performance we have more specifically focused our analysis on 15 stations. This subset is chosen to cover different coastal environments^49^ and therefore surge characteristics (as further shown in Fig. 5).

#### Predictor variables from high resolution climate reanalysis data

The ERA5 dataset of ECMWF provides a global atmospheric reanalysis with a spatial resolution of 0.25° and an hourly temporal resolution. In order to predict surges, we extract atmospheric variables at a centered box of 1.25° around the station (i.e. 5 × 5 cells). In total, five variables are used as predictors: the mean sea level pressure (MSLP), the hourly gradient of the MSLP (Δ MSLP) the meridional and zonal wind 10-m wind components (U and V), and the wind speed magnitude. As with the surge data, the ERA5 data have been detrended by removing the inter-annual mean variability.

### Neural network models and skill metrics

Four different types of NN models are set up and trained to predict surges: an Artificial Neural Network (ANN), a Long Term Short Term Neural Network (LSTM), a Convolutional Neural Network (CNN), and a Convolutional LSTM (ConvLSTM), which is a combination of the latter two. In this section, we provide an overview of their specific features and selected architecture. For more detailed information, the reader is referred to the accompanying references shown in the following section.

The ANN, the most general form of NN, has been extensively applied in various fields of science to capture nonlinear processes^31^. The LSTM is a derivation of the Recurrent Neural Network (RNN) in that it captures sequence-to-sequence patterns in their internal state as memory, but has advantages over the conventional RNN as they can selectively store long term information^31,36,50,51^. The CNN is a class of NN models that works well in capturing spatial features, shapes and texture due to its shared weight architecture and is thus often applied for image recognition purposes^31,33,52,53^. We also apply a ConvLSTM in order to capture spatiotemporal information. The ConvLSTM combines sequence-to-sequence learning with convolutional layers and emerges in current studies with promising applications in predicting spatiotemporal information^54^.

All the NN model types use the same input data, i.e. the predictor variables, albeit in different formats and their architecture is shown in Supplementary Figure S4. They have a similar overall architecture, constructed from a series of layers made of neurons connected to each other. The first layer (called the input layer) contains the input data, i.e. the predictor variables. This input layer is connected to one or multiple hidden layers and finally connected to an output layer, which provides the surge predictions. Information between neurons in consecutive hidden layers is transferred through weighted connections, summed with a bias and scaled using a so-called activation function before being transferred to the neurons of the next layer. All parameters defining the model architecture (e.g. number of neurons, number of hidden layers) and learning process (e.g. choice activation function, subset size of training data (batch size)) are referred to as hyperparameters^51^. Other important hyperparameters often applied to prevent overfitting are the dropout percentage of the neurons in the last layer (dropout) and layer weights regularizer functions (kernel regularizer)^55^.

We use the Python hyperparameter optimization package SHERPA^56^ to find the number of neurons, the dropout rate, the regularizer factor l2 in the kernel regularizer function, the number of filters for the CNN/ConvLSTM, and the batch size leading to the lowest mean absolute error (MAE) between predicted and observed surge levels. To do so, we apply a random search optimization with a maximum of 100 trials for each NN at the 15 stations selected (see Fig. 1). The settings leading to the lowest loss across all 15 stations have been used as default settings for the NN models for all stations. This resulted in the following hyperparameters: 24 filters (for the CNN and ConvLSTM), 48 neurons (for the CNN and ConvLSTM, this applies for the last fully connected layer), a dropout value of 0.1, l2 weight regularizer factor of 0.001 and a batch size of 10 days (240 hourly timesteps).

Depending on the NN model type, the input layer is connected to the following hidden layer:

- *ANN* A fully connected layer with an l2 kernel regularizer.
- *LSTM* a stateless LSTM layer with a hard sigmoid recurrent activation function.
- *CNN* a 2D convolution layer. Each filter has a kernel size of 3 × 3 with same padding and the convolution step is followed by a max-pooling layer with a kernel size of 2 × 2.
- *ConvLSTM* a 2D convolution layer following a stateless LSTM layer with a hard sigmoid recurrent activation function. Each filter has a kernel size of 3 × 3 with same padding and the convolution step is followed by a max-pooling layer with a kernel size of 2 × 2.

All of the NN models are activated using the ReLu activation function^57^. In the cases of the LSTM and ConvLSTM, a hard sigmoid function is used for the recurrent activation^58^. The last hidden layer is a fully connected layer with an l2 weight regularizer and after that dropout layer is added. We select the Adam optimizer algorithm (learning rate of 0.001) for the learning rate optimization algorithm and train the NN model to minimise the MAE, the selected loss function, between observed and predicted surge. The output layer, with one node only, represents the predicted surge levels. Because the four NN have different specifications, flattened input data without spatial relationships are fed to the ANN and LSTM. For the CNN and ConvLSTM input dimensions with spatial relationships between grid cells are fed into the NN. Note that for the NN with convolutional layers (CNN and ConvLSTM) the spatial input dimensions are relatively small in relation to the kernel size due to the spatial resolution of the ERA5 data and computational constraints as hourly data is used as temporal resolution in this study. Figure S4 shows the architecture and input dimensions of the different NN model types used in this study. Additionally, we provide the NN models in the Supplementary Data, whose architecture and hyperparameters can be viewed using a NN visualizer app, such as netron.app.

**Figure 2:**
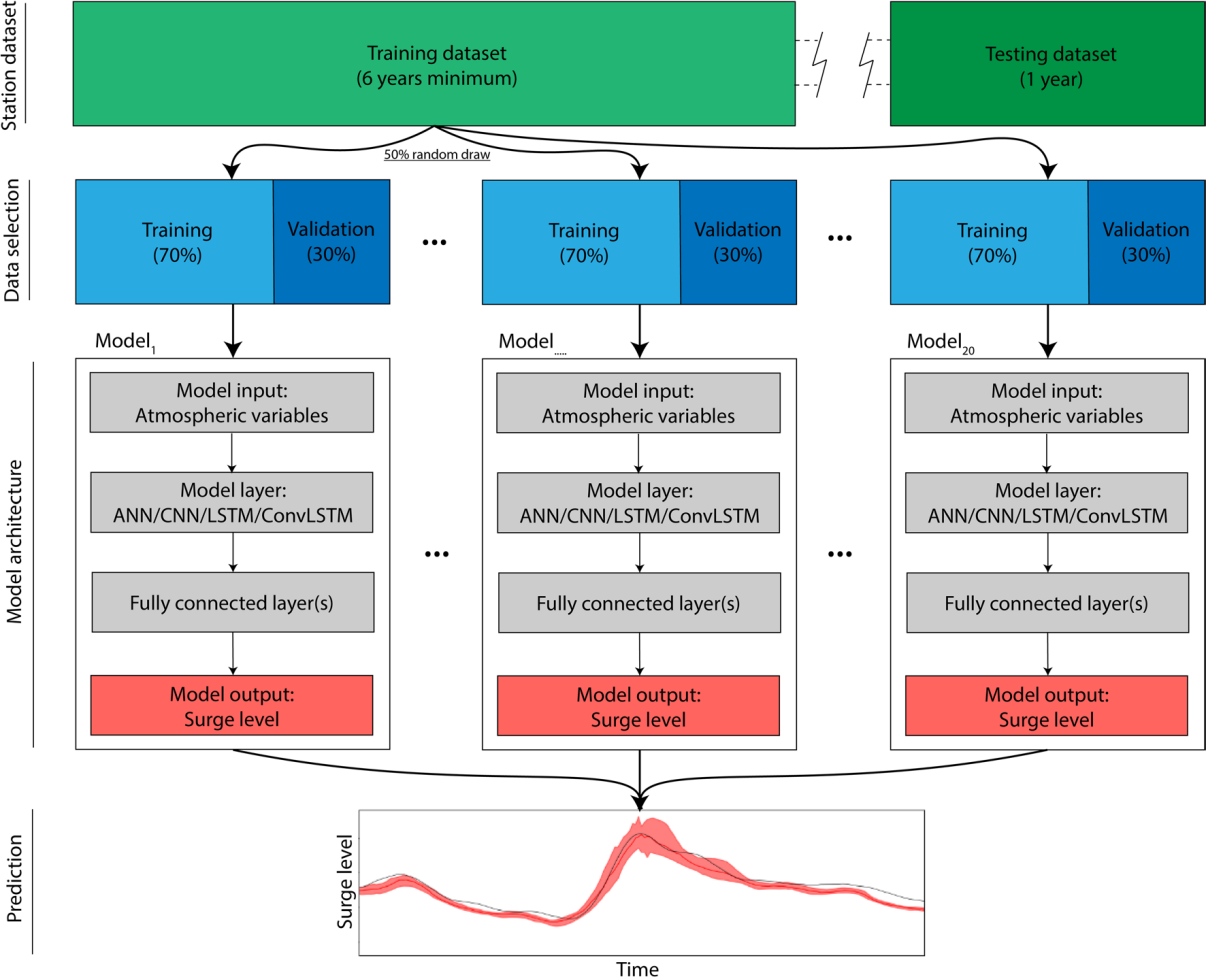
Overview of NN model ensemble and selection of data.

Figure 2 shows an overview of our model chain and ensemble prediction methodology. We partition the predictor and predictand datasets to make the distinction between three phases: training, validation and testing. Training and validation phases are repeated iteratively to update and tune the model parameters between each iteration, i.e. the so-called epoch. We set the maximum number of epochs to 150 but stop the training phase if no improvement in the loss is detected from the three previous epochs. The testing phase, using data excluded from the training and validation dataset, provides an unbiased evaluation of the model performance for the model parameters selected. We use the most recent year without gaps (365 consecutive days). The rest of the data are allocated into subsets of training data and validation data without gaps. In order to provide probabilistic predictions of surge levels, we use random subsets from this data to fit a model and repeat this operation 20 times to construct an ensemble of 20 models for each NN type similar to Barbarossa et al.^59^ and Bruneau et al.^11^ as follows. Between the models we use different subsets of the training data while keeping the same model configuration. For each individual model of the ensemble, we randomly sample 50% of the rest of the data and train the model by using 70% of the selected data as training and 30% to validate the model, both randomly selected. For the LSTM and ConvLSTM, the random sampling is performed on batch-sized consecutive sequences without gaps from the time series. Between the epochs, we shuffle the training data (for LSTM and ConvLSTM we shuffle the batch-sized sequences). This ensemble prediction methodology has been set up as a nowcast structure, in which the models are trained on historical data, but can be applied on new data from the same datasets.

The implementation of the NN model types is done with the Python package Keras^60^, which uses Python package Tensorflow as a backend^61^. Predictors and predictand data are transformed by subtracting the mean and dividing by the standard deviation based on the training data only and these normalization parameters are stored. The predicted surge levels from the NN models are back-transformed using these parameters. We also tested other transformations for three stations (such as the scaling of each variable based on local maximum and minimum between 0 and 1 and the Yeo-Johnson transformation), but this did not lead to significant improvements in performance. Data transformation is carried out using the Python package scikit-learn^62^.

#### Probabilistic performance and skill metric

Since the NN models provide an ensemble of surge predictions, we select the Continuous Ranked Probability Score (CRPS) for the NN performance metric, a metric that can account for probabilistic predictions^63^. Due to the absence of explicit observational uncertainty, the CRPS is more suitable than other metrics such as relative entropy (KL divergence), and is a widely used metric within hydrology and coastal science for probabilistic prediction^11,64,65^. The CRPS averages the difference between the observed and predicted cumulative distribution of the surge across all time steps and for deterministic forecasting it reduces to the MAE:

1 $$ CRPS = \frac{1}{N}\mathop \sum \limits_{t = 1}^{N} \mathop \smallint \limits_{ - \infty }^{\infty } \left[ {P_{t} \left( x \right) - P^{o}_{t} \left( x \right)} \right]^{2} dx $$

where N is the number of time steps in the testing year (365 × 24 h), $P_{t} \left( x \right)$ is the predicted stepwise cumulative distribution function (CDF) at time t = i and $P^{o}_{t} \left( x \right)$ is the observed CDF at time i of the surge $x$. Because the observed time series is deterministic, $P^{o}_{t} \left( x \right)$ reduces to the Heaviside function such that $P^{o}_{t} \left( x \right)$ = 0 below the observed value and $P^{o}_{t} \left( x \right)$ = 1 at the observed surge value and above at time $t$.

The CRPS is a negatively oriented score and can vary from 0 to ∞, with a value of 0 indicating a perfect deterministic prediction. We report the CRPS in all figures in centimetres. High CRPS scores reflect that the surge distribution is not properly predicted: a wide ensemble spread of predicted surge will capture the observed surge but does not reflect any model skill. On the other hand, a low spread in the model predictions and a model bias will also result in a high CRPS score, as the ensemble will fail to capture the observed surge. In order to better understand the behaviour of the ensemble prediction, the CRPS can be decomposed^63^ as:

2 $$ CRPS = RELI + CRPS_{pot} $$

where $RELI$ is the reliability component, which indicates whether the distribution of the predicted surge has similar statistical properties to the observed time series, and $CRPS_{pot}$, the potential CRPS, denotes the CRPS when the ensemble forecasts are perfectly reliable ($RELI$ = 0, also a negatively oriented metric). In order to decompose the CRPS into the reliability and potential component, we use the crpsDecomposition function from the R package verification^66^. The potential CRPS is further decomposed into:

3 $$ CRPS_{pot} = U - Resol $$

where the uncertainty component, $$U$$, represents the CRPS if only a climatological probabilistic prediction is available, and the resolution component,$$ Resol$$, evaluates the improvement of the NN model ensemble predictions to the average ensemble spread and the observed outliers. A resolution value higher than zero indicates that the model ensemble adds some value compared to the probabilistic climatology distribution. By definition, the climatological probabilistic prediction has no model skill and a perfect reliability. We derive the climatological probabilistic prediction using a stratified sampling approach of the observed surge^67^. We remove noise and spurious effects by calculating a centered moving average with a Gaussian filter and a window size of 30 days^68^ of the surge used in the training dataset and weighted with the following equation:

4 $$ w = \frac{1}{2\Pi \sigma } $$

where $\sigma$ is the standard deviation expressed in time steps and set to 72 h. Next, we rank the hourly surge levels for each month of the year and draw 20 equidistant samples to construct the cumulative distribution function of the climatology. These 20 samples represent the predicted ensemble from the probabilistic climatology predictions and are used to calculate the uncertainty component for the CRPS value of the testing year.

Comparing CRPS values between stations can be difficult to interpret because similar CRPS values does not necessarily indicate a similar model skill. In stations with a larger variability, the uncertainty component is larger and the CRPS becomes larger in value and therefore seemingly worse. To correct for this effect, we scale the CRPS value with the uncertainty component (see Eq. 3). We select the best NN model type as the one with the lowest CRPS value, $CRPS_{best}$ , and calculate the skill gain of each NN model type by normalizing the CRPS value with the CRPS value of the climatological distribution with the following equation:

5 $$ CRPSS = \frac{{U - CRPS_{best} }}{U} \times 100 $$

where CRPSS is now a dimensionless and positively oriented indicator of the skill gain compared to that of the reference ensemble predictions from the probabilistic climatology distribution^69^. The ratio is multiplied by 100 to represent the CRPSS values as a percentage. The CRPSS can vary from − $\infty$ to 100%, with 100% representing a perfect prediction and values higher than 0 indicating a better performance than the probabilistic climatology forecast.

### Assessing the influence of NN architecture and predictor variables

The selection of the hyperparameters, predictor variables, and spatial footprint considered around each station have been tuned following an optimization with a simple design rationale for 15 stations, as explained previously. The best settings across the different NN types have been used as default settings for the NN models for all stations. Here, we explore the effect on model performance for the 15 selected stations in response to increasing the spatial footprint of input data, number of predictor variables, and NN design architecture for the four NN types. We use the default settings and perform a sensitivity analysis for each of them. First, we test the influence of the size of the spatial footprint on the performance of the NN models and gradually increase the gridded box centred around the station by 0.5° degree resolution (0.25°, 0.75°, 1.25°, etc.). Second, we gradually increase the number of predictor variables in the following order: MSLP, wind speed magnitude, U and V, gradient of MSLP, and finally the quadratics of U and V. By doing so, we show the sensitivity of adding more atmospheric predictor variables on model performance. Last, we perform a hyperparameter optimization but focussing on the architecture of the NN model (hidden layers, neurons and filters). For this optimization we opt for using an optimization algorithm instead of a one-by-one sensitivity analysis, because of the large number of combinations and showing the effects of optimizing on one model for an ensemble. The random search algorithm with a maximum of 100 trials optimizes the number of hidden layers (1, 2, 3, 4, 5), Neurons (24, 48, 96, 192), and filters (8, 16, 24).

## Results

### Global performance and skill of the NN models

**Figure 3:**
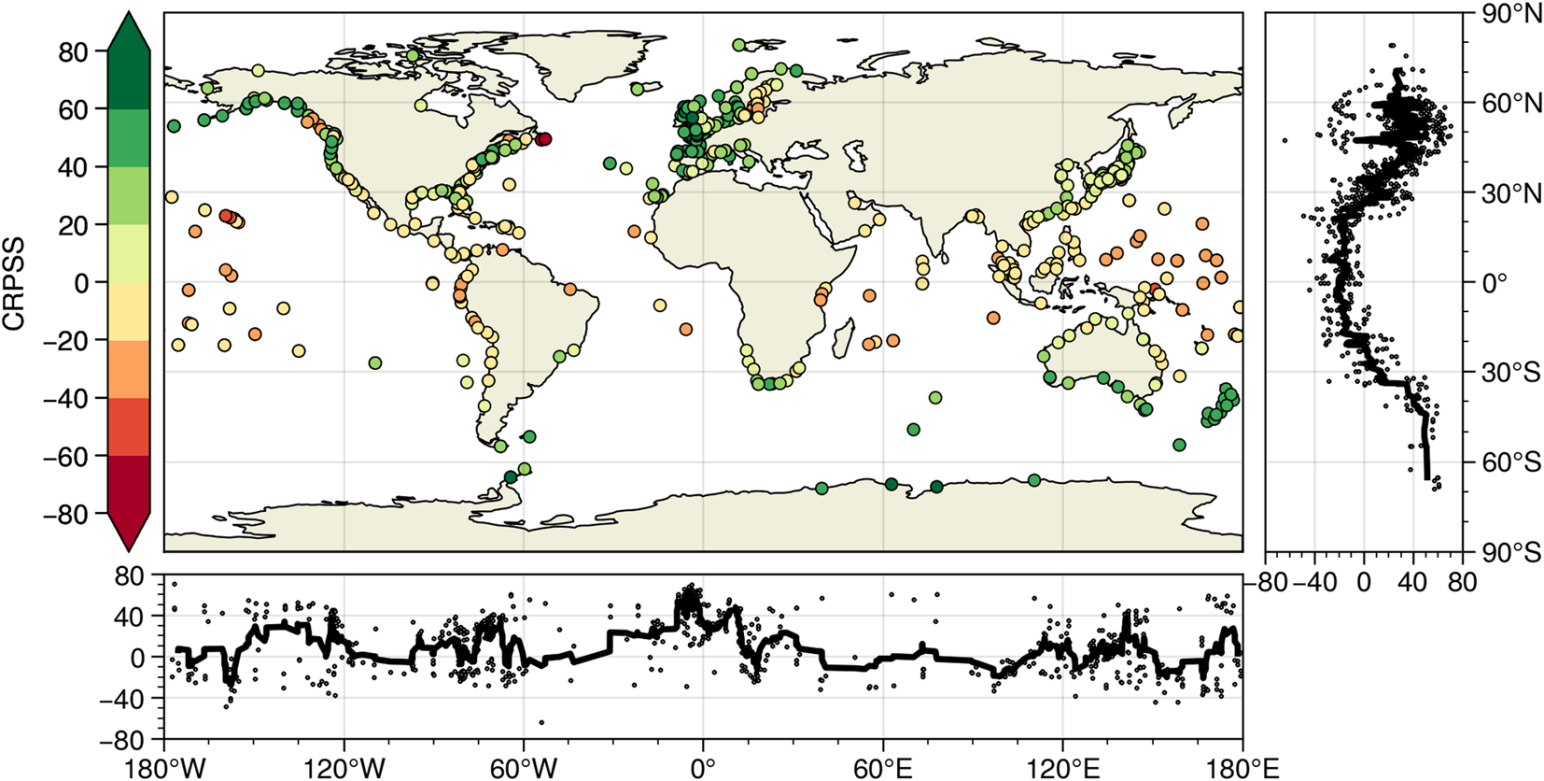
Highest CRPSS percentage obtained of all NN models per tide station. Meridional and zonal plots present the CRPSS values with a centred moving average of 10 stations.

The CRPSS for each station for the best NN model (i.e. the one with the lowest CRPS) is shown in Fig. 3. A positive (negative) CRPSS indicates that the NN model has better (worse) predictive skill compared to our reference model, i.e. the probabilistic climatology ensemble. We observe clear spatial patterns in high and low model skill. Stations along the coast of West Europe, East Asia, New Zealand, Southern Australia, Southern Africa, and parts of North and South America generally perform well, with a CRPSS generally higher than 40%. However, stations close to the equator perform poorly, with negative CRPSS scores. These spatial patterns of model skill performance are observed for all NN model types (Figure S5) and also found in large scale hydrodynamic models^8^. This indicates that these differences do not necessarily stem from the model type applied but rather point towards more general challenges in capturing surge variability in the tropics.

We investigate the CRPS decomposition to better understand the NN model performance. Similar spatial patterns are observed for the uncertainty and the resolution component (Figure S6b,d, respectively), with lowest resolution scores and lowest uncertainty scores at stations near the equator and higher values in mid- to high latitudes. The resolution component of the CRPS, Figure S6d, is positive for all stations except two locations located southeast of the Newfoundland island, Canada. The positive resolution values indicate that NN models improve the surge ensemble predictions from our agnostic climatological probabilistic prediction. This does not however guarantee a better model skill. Negative values of the CRPSS can be interpreted as that the overall distribution of observed values is better captured by the distribution of the climatological mean than the NN model predictions. Nonetheless, this shows that there is value, albeit limited, in using a NN and indicates an improvement gained by the NN to better capture the average ensemble spread and behaviour of observed outliers.

**Figure 4:**
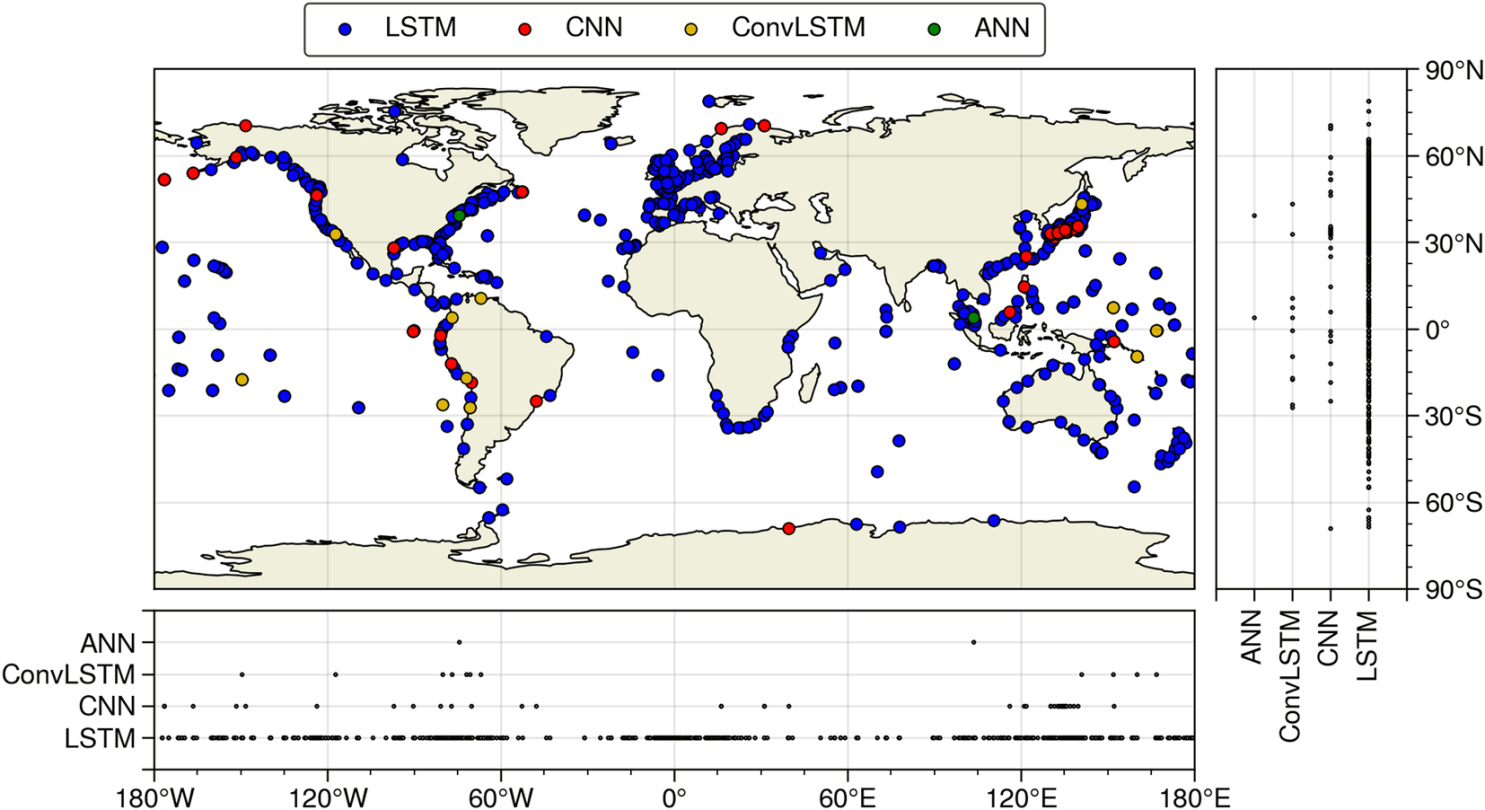
NN model type from the highest CRPSS value shown in Fig. 3. For readability, we display the models in the following order LSTM → CNN → ConvLSTM → ANN.

The best-performing NN model type per station is shown in Fig. 4. Although the differences between NN model types are marginal (Figure S5), the LSTM results in the best performance for the majority of the stations (92%). Regions in Europe, Africa, Australia, the Pacific, and the U.S. almost all show the highest CRPSS values for LSTM. The only region that shows a clustering of best performance for the CNN is the southeast of Japan. Convolutional LSTMs show the best performance for a few locations along the east coast of South America and for five islands in the Pacific Ocean. The ANN performs best for only two locations (Atlantic city, USA; and Kuantan, Malaysia). Comparing the CRPS decomposition between the NN model types shows that for almost all stations the reliability score is the lowest for the LSTM. This indicates that the probabilistic forecast from the LSTM has the closest agreement to the distribution of the observed values (Figure S7). Similarly, since the uncertainty component of the CRPS is the same for all NN models (by definition), this implies that the resolution is highest for the LSTM at most stations. The LSTM is best in distinguishing types of events and their different distributions of expected surge. In terms of computational time, the LSTM is almost as fast as the ANN, about 1 min per model on a Bullx system supercomputer, while the CNN and ConvLSTM take on average three and 18 times longer than the ANN, respectively.

#### Local performance at selected tide stations

**Figure 5:**
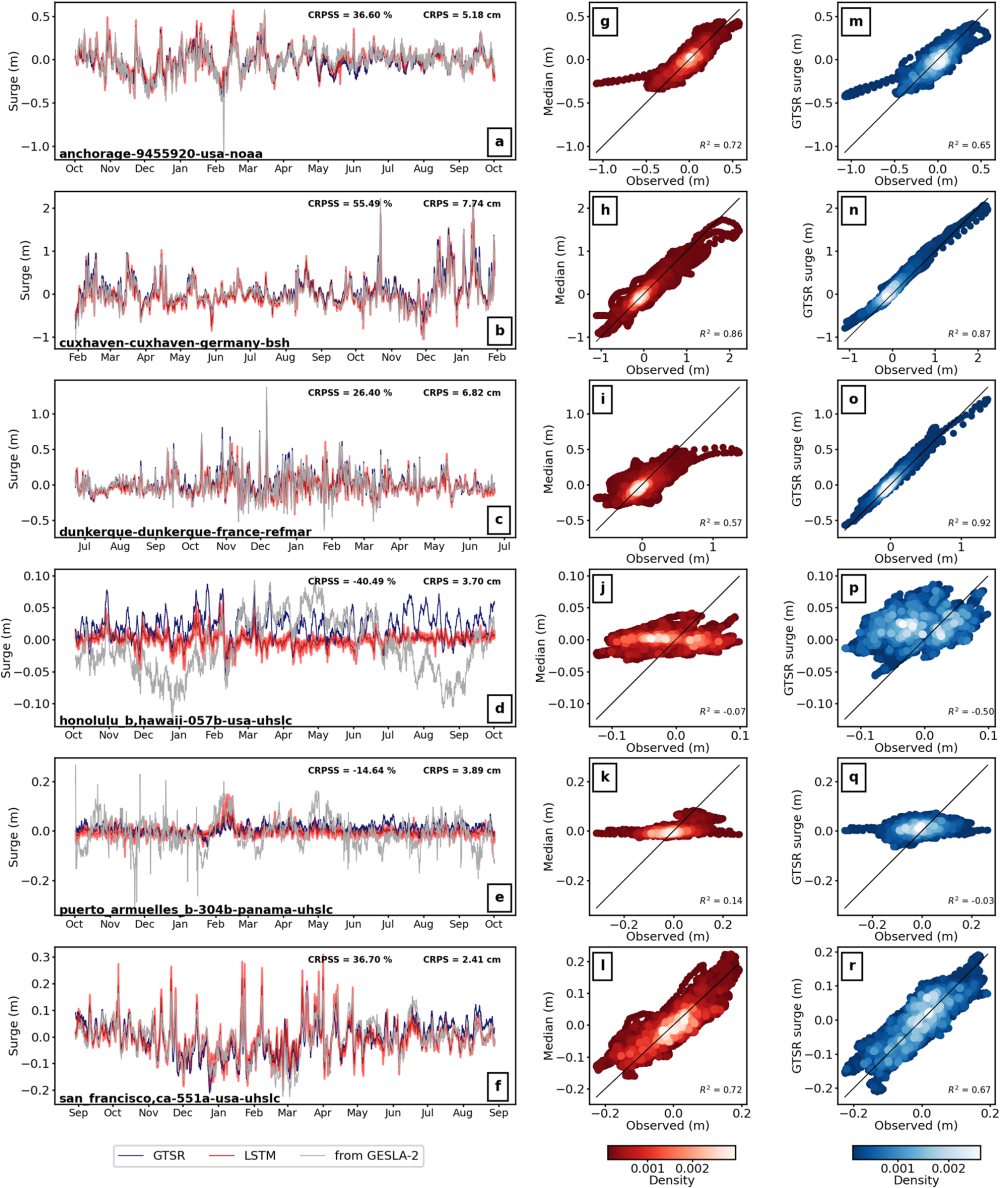
(**a–f**) Hourly surge predictions for the testing year from the LSTM model, observed and surge from the Global Tide and Surge Model forced with ERA5 and (**g**–**l**) scatter plot of the median from the predicted ensemble with the observed surge and (**m**–**r**) with the GTSM surge.

Figure 5 shows the ensemble surge time series predicted from the best NN model type at six of the fifteen selected locations with the observed time series for the testing year. The rest of the stations are shown in Figure S8 and S9. For all 15 stations, the LSTM is the best-performing NN model except at Callao, where the CNN model shows the best skill (CRPSS from LSTM: − 13.41%; CRPSS from CNN: − 13.10%). We note that an inter-station comparison based on the CRPS metric alone would have been misleading and incorrect. As shown in Fig. 5, stations with the best (highest) CRPSS score do not necessarily have the best (lowest) CRPS score. This is because the uncertainty component of the CRPS, representing a climatological probabilistic prediction, greatly differs per location (as indicated by the difference in the range in scale of the y-axis). Instead, using the CRPSS as our comparison metric normalizes for these differences in climatology.

In Fig. 5, a positive CRPSS is obtained at four stations, with the highest value of 55% for the Cuxhaven station. At these stations, the general temporal evolution of the surge is well captured and there is close agreement between observed and predicted time series although extremes are often underestimated. We extract the median from the probabilistic predictions to calculate the coefficient of determination, R^2^, with the observed surge levels. R^2^ range between 0.57 at Dunkerque up to 0.86 at Cuxhaven. At stations with a negative CRPSS (Honolulu: − 40.49%; Puerto Armuelles: − 14.44%), we observe that the NN cannot capture low frequency variations of the observed time series that dominate the overall variability. As a result, the lower model skill is lower than ensemble predictions based on the climatology distribution. These low frequencies probably driven by mean sea level variations, independent of atmospheric conditions, and therefore acts here as noise. This effect is also visible for other stations shown in Supplementary S8 and S9 such as Zanzibar (CRPSS: − 22.46%), Lord Howe (CRPSS: − 14.15%) or Callao (CRPSS: − 13.10%).

We compare these predictions with the surge time series obtained from the Global Tide and Surge Model (GTSM), a global hydrodynamic model simulating tides and surge forced with ERA5^8^. There is a close agreement between the NN skill and the results obtained with GTSM. Comparable R^2^ values to the NN are found at the stations with a positive CRPSS score and lower R^2^ values for negative CRPSS, see Fig. 5m–r, S8 and S9. At the stations of Cuxhaven and Dunkerque, GTSM provides very high agreement with observations, with R^2^ of 0.87 and 0.92 respectively compared with 0.86 and 0.57 for the LSTM. Averaged across all the 15 stations, we find an average R^2^ value for the LSTM (GTSM) of 0.69 (0.67) for stations with a positive CRPSS stations and 0.08 (− 0.16) for negative CRPSS. More generally, the close performance between GTSM and LSTM tends to confirm that poor model skill observed is the result of a lower frequency signal present in the observed time series but not driven by meteorologically driven processes since it is also absent from the GTSM surge time series.

### Assessing the influence of NN architecture and predictor variables

To explore ways to further improve the performance of the ensemble models, we evaluate the skill of the NN model and the complexity of the model input by increasing the number of predictors together with increasing the size of the spatial footprint by 0.5 degree at a time. The spatial footprint is increased from 0.25 degrees (1 × 1 cells where the tide station is located) up to 6.25 degrees (13 × 13 cells) centred box around the station. Furthermore, we perform a hyperparameter optimization allowing more hidden layers (up to 6) in our model architecture together with an optimization of the number of neurons (24, 48, 96, 192) and filters (8, 16, 24).

#### Influence of predictor variables

**Figure 6:**
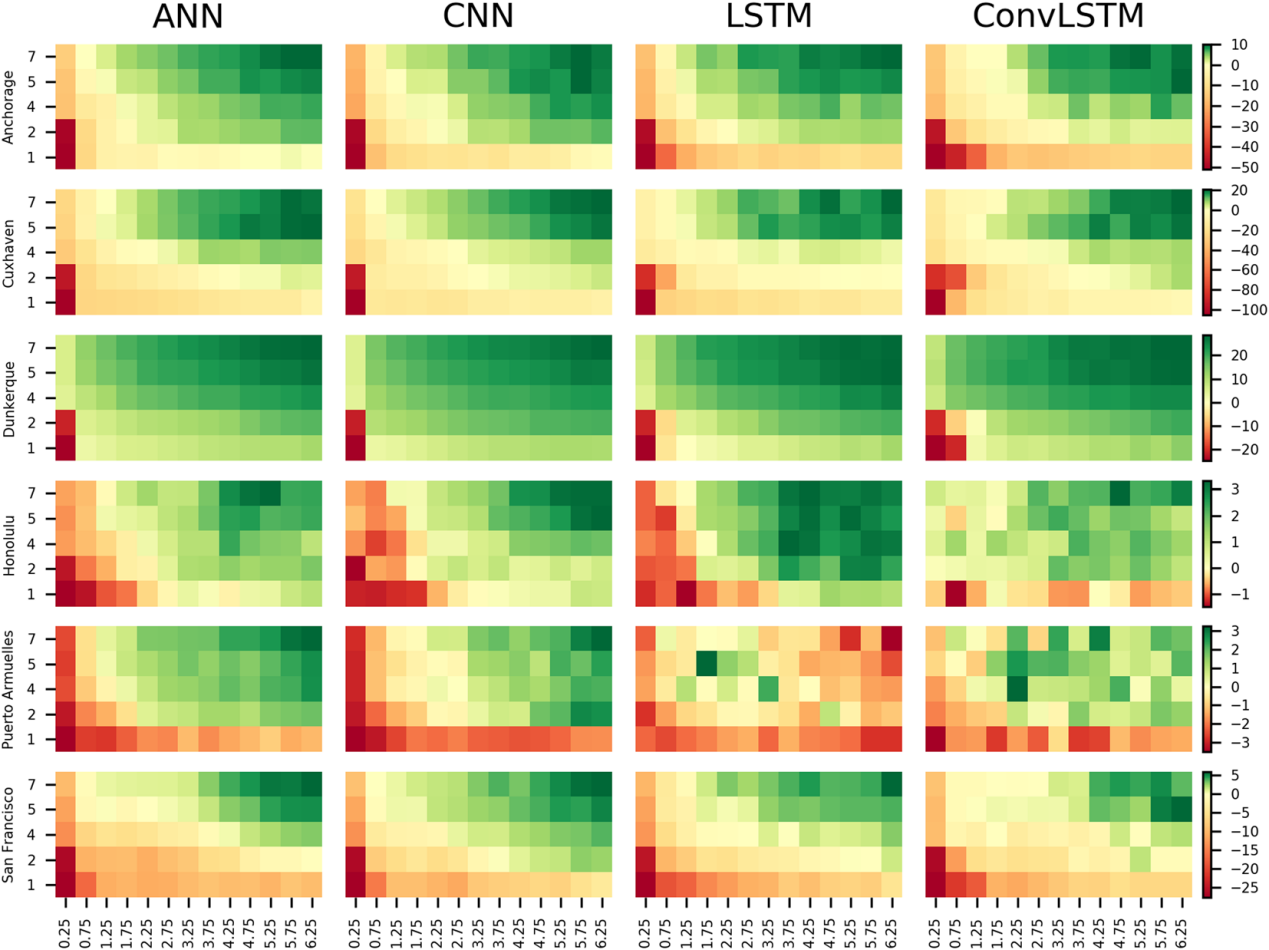
CRPSS value of the NN model ensemble for increasing number of predictor variables and spatial footprint size. On the horizontal axis the spatial footprint is depicted and on the vertical axis the number of predictor variables are depicted with cumulatively 1 (MSLP); 2 (wind magnitude); 4 (U and V); 5 (gradient of MSLP); 7 (quadratic of U and V).

Figure 6 shows the CRPSS value obtained when increasing the number of predictor variables (y-axis) and the spatial footprint size (x-axis) for the NN for the six locations used throughout this study. Additionally, the same plots for the nine other locations are shown in supplementary Figure S10. The CRPSS improves when enlarging the spatial footprint size for almost all stations and NN types. Moreover, stations that show an increase in CRPSS from a negative to a positive score (from dark red to green) when increasing the number of predictor variables or spatial footprint show the ability to learn of the NN at that location. Increasing the spatial footprint from 0.25 degree resolution to 0.75 degree shows the largest increase in CRPSS value (9.5% on average for all 15 stations). After a spatial footprint of 2.75 degree, the CRPSS improves on average with 0.55% for each 0.25 degree increase of spatial footprint. Between the different NN types, we see that the performance of the CNN and ANN has the largest ability to learn when increasing the spatial footprint as the CRPSS of these NN types improves the most. Furthermore, we see that for stations that have a lower or negative CRPSS like Puerto Armuelles (also Ko Taphao, Lord Howe and Zanzibar in Figure S8) the learning rate of LSTM increases/decreases sporadically when increasing the spatial footprint. Additionally, the average ensemble model spread increases and leads to a lower CRPSS value. The spread and uncertainty of the ensemble models for the LSTM and ConvLSTM increases when adding complexity to those stations. Due to this larger spread and uncertainty in the predictions, we argue that the LSTM and ConvLSTM do not benefit much from an increase in input or architecture complexity. Next to this, we find no clear difference in learning when increasing the spatial footprint when splitting the stations into stations that are prone to be hit by (extra) tropical cyclones and stations that are not (Figure S11).

When increasing the number of predictor variables, we see that the best performance is gained when using the 5 (MSLP, gradient of MSLP, U and V, and wind magnitude) or 7 (same as 5 but the quadratic U and V are added; hereafter referred to as U^2^ and V^2^) predictor variables. Averaged over all 15 stations, we see that when including the wind magnitude predictor to the MSLP in the 2 predictor setup, CRPSS performance increases the most with an average of 5%. For the other predictors (U and V, gradient of MSLP, and U^2^ and V^2^) performance increases on average of 4.2%, 2.3%, and 0.5% respectively. Highest performance is reached when including the gradient of the MSLP and U^2^ and V^2^ in the 5 and 7 predictor setup respectively. Although the CRPSS shows an improvement in model performance for all 15 stations, the computation time increases a lot when enlarging the input dimensions (Figure S9). Compared to the setup of the global runs and depending of the computation architecture, the computation time, in our case, increases up to 15 times: from 0.3 and 4.8 min for the global settings runs to 3.1 and 65 min for the fastest and slowest NN which are LSTM and ConvLSTM respectively. Of course, this is just an indication as performance is dependent on layer configurations and hardware. In our case, details of the thin nodes we used can be found here (https://userinfo.surfsara.nl/systems/cartesius/description).

#### Influence of NN architecture

Next to increasing the number of predictor variables or spatial footprint, we assess the influence of locally optimizing the number of hidden layers, neurons and filters (for the CNN and ConvLSTM). For the global results, we used a fixed NN design with only one hidden layer of each type and hyperparameters of 48 neurons and 24 filters for the convolutional layers (for the CNN and ConvLSTM only). We compare the performance of the optimized NN models from a local hyperparameter optimization on the number of hidden layers, neurons, and filters with a random search algorithm with that of the models from “Global performance and skill of the NN models” section. At each of the 15 tide stations, the optimization minimizes the MAE using the whole training dataset.

Table 1 shows the results of this hyperparameter optimization for the 15 stations selected. Across all model types, we see that a local optimization leads to a different selection of hyperparameters between stations. In comparison to the CNN and ConvLSTM, we observe a larger spread in the number of optimal hidden layers for the ANN and LSTM ranging between 1 and 5 hidden layers. On average, the CNN requires 3 or more hidden layers (median: 4) and the ConvLSTM centres around 2 or 3 hidden layers (median: 2). The number of filters is preferably 24 for CNN and ConvLSTM, while the latter also sometimes favours a lower number of filters.

**Table 1:** Results of the NN architecture hyperparameter optimization.

Stations	CRPSS				Change CRPSS				Change hit rate				Hidden layers				Neurons				Filters	
	ANN	CNN	LSTM	ConvLSTM	ANN	CNN	LSTM	ConvLSTM	ANN	CNN	LSTM	ConvLSTM	ANN	CNN	LSTM	ConvLSTM	ANN	CNN	LSTM	ConvLSTM	CNN	ConvLSTM
Anchorage	29.4	40.5	36.8	33.9	0.7	6.2	0.2	− 0.2	1.2	17.5	1.9	5.0	3	4	5	2	48	96	48	96	24	16
Boston	31.8	37.7	37.0	33.7	1.7	5.5	− 0.9	0.1	2.3	8.9	− 2.6	− 3.6	4	4	2	2	96	96	24	24	24	16
Callao	− 14.5	− 3.8	− 10.5	− 13.2	1.2	9.3	2.9	4.1	3.2	35.4	5.0	3.0	2	4	4	1	24	48	96	24	24	8
Cuxhaven	51.7	56.5	53.2	48.9	3.7	11.7	− 0.5	1.5	− 1.9	12.0	3.7	− 3.9	4	4	4	2	48	24	192	24	24	24
Dakar	− 20.6	− 6.9	− 11.4	− 19.6	− 0.2	11.2	0.9	− 1.0	0.2	19.7	4.0	1.1	1	5	1	5	48	192	192	96	24	16
Darwin	− 1.5	5.5	1.5	− 7.3	0.7	4.3	− 0.6	− 6.2	− 0.2	11.0	0.3	− 2.7	4	5	2	3	96	48	48	96	24	16
Dunkerque	34.4	41.7	40.9	36.2	15.9	21.7	14.5	15.4	0.8	12.1	1.7	2.6	2	3	1	2	24	24	96	192	24	24
Honolulu	− 45.4	− 40.8	− 41.5	− 43.0	− 0.3	4.5	− 1.0	− 0.4	− 0.3	6.6	0.2	0.0	1	5	3	1	96	192	24	24	24	8
Humboldt	35.0	41.9	41.3	35.9	− 0.1	4.9	1.7	− 1.2	0.3	17.0	9.0	0.2	2	4	5	2	48	24	192	24	24	24
Ko Taphao	− 35.2	− 9.9	− 26.2	− 30.1	− 0.4	23.6	− 0.1	1.8	0.5	23.1	0.0	0.1	3	5	2	2	24	48	96	48	24	8
Lord Howe	− 18.9	− 18.3	− 14.6	− 17.4	− 0.4	1.5	− 0.4	− 1.5	− 1.1	4.0	3.4	− 0.8	1	4	4	1	24	96	192	48	16	8
Puerto Armuelles	− 20.6	− 10.7	− 21.8	− 23.2	0.0	6.2	− 7.1	− 4.2	0.1	30.4	− 5.0	− 3.9	3	3	5	3	24	48	192	192	24	16
San Fransisco	30.4	35.6	36.2	34.6	0.3	4.4	− 0.4	1.0	− 0.7	15.1	− 3.4	3.3	2	5	1	3	24	96	192	48	24	24
Wakkanai	33.7	41.0	38.7	34.2	0.6	4.3	− 0.8	− 1.2	2.3	11.6	− 1.8	2.0	2	3	2	4	48	96	96	48	24	16
Zanzibar	− 31.9	− 22.0	− 28.0	− 29.0	0.3	6.1	− 5.5	− 1.1	0.8	14.3	− 5.5	− 1.5	1	4	5	2	48	24	48	24	24	16
Mean/median	3.9	12.5	8.8	5.0	1.6	8.4	0.2	0.5	0.5	15.9	0.7	0.1	2	4	3	2	48	48	96	48	24	16
Standard deviation	32.2	30.6	32.4	32	4.1	6.4	4.7	4.8	1.3	8.5	4	2.8	–	–	–	–	–	–	–	–	–	–

The change in hit rate is expressed in percentages increase of model ensemble ability to capture observed value within the ensemble spread. The summary statistics shown are mean for CRPSS, change in CRPSS and change in hit rate, and the median for the hyperparameters.

In Table 1, we also report the CRPSS obtained from the ensemble predictions using these optimized settings and the absolute change in CRPSS, i.e. the difference between the CRPSS obtained here and the CRPSS from the previous NN model applied in “Global performance and skill of the NN models” section. We see that the change in CRPSS is positive for all stations with the CNN and up to an increase of more than 20% for Dunkerque and Ko Taphao. However, this increase in CRPSS for Ko Taphao does not indicate a meaningful ensemble model improvement as the CRPSS is still negative. For instance, ensemble model predictions for Ko Taphao could become better at generating random predictions that together look like climatology. For these stations, we also see an increase in the hit rate defined as the percentage of hourly time steps the NN ensemble spread captured the observed surge value. This therefore suggests an increase in the ensemble spread that is capturing the signal.

For the other NN types, the change in CRPSS is generally smaller, showing a marginal gain from applying more complex NN models. The local optimization even results in a decline in CRPSS value for some NN model types and stations, e.g. the LSTM and ConvLSTM for Puerto Armuelles. This decrease in performance from the local optimization denotes a mismatch between the hyperparameter optimization on the MAE on the *whole* training data and the performance obtained from *ensemble* predictions. This indicates that optimizing for a deterministic prediction does not necessarily lead to a better probabilistic prediction. The change in hit rate frequency indicated in Table 1 shows that NN models that do not improve in CRPSS can improve in hit rate frequency by increasing the spread between members. Each member however, will be less representative of the others and of the surge progression in reality, leading to a lower CRPSS value than original.

## Discussion

This study provides the first application of four NN model types for hourly surge predictions at the global scale and explores the capabilities of NN models. Unlike previous data-driven studies, we benchmark the performance of the NN models developed in this study against simple probabilistic predictions based on climatology to further understand spatial performance patterns. We also provide, for the first time, a quantitative assessment of the role of the network complexity, the number of predictor variables considered and the spatial extent considered around each location with respect to the model performance.

Overall, the results found in this study are in line with previous studies from Cid et al.^19^, Tadesse et al.^10^, and Bruneau et al.^11^, which are to our knowledge the only other studies that have looked at either daily or hourly surge predictions from data-driven models at the global scale. Focusing on 15 stations, we found very similar performance between the GTSM hydrodynamic model^23^, and the LSTM. An important similarity across all these studies is that model skill increases from low to high latitude, where climatology shows that there is more variability in surge levels. This finding is consistent even though model setups and the selection of predictor variables differ across all these studies. This therefore seems to indicate more general challenges in surge predictions, which are summarized next.

In order to predict surges, we selected the predictor variables from atmospheric variables that are known to be the main drivers of surge^10,70^. Our analysis of model skill however suggests that other sea level components are still present in the observed time series that cannot be captured by our predictor variables. This effect is particularly visible in the tropics where we find a consistently low model skill, similar to other studies^10,19^. Surge variations in tropical regions are often characterized by a small variance, in the order of a few centimeters. In this case, errors in the tidal decomposition can introduce spurious noise of the same magnitude or more in the non-tidal residual time series that can have a relatively large influence on the time series and the CRPS. In tidally dominated coastal environments, applying a recursive Chebyshev Type II low-pass filter instead of a moving average might better help remove remaining transitory signal in the non-tidal residuals and isolate the high frequency signal in the observed time series^44,48^. Moreover, other processes not linked with atmospheric variability can still be present in the observed time series. Steric components (driven by seasonal changes in salinity and thermal expansion), geostrophic currents (due to oceanographic pressure gradients), and river discharge levels in estuaries and deltas can have a similar or larger influence on non-tidal water level variability than surge levels^3,4,9,71–76^. Therefore, one could instead add additional predictor variables to cover these different processes. Examples of additional predictor variables available at the global scale that have been applied in other data-driven studies are sea surface temperature, accumulated precipitation, significant wave height, and peak periods^9–11^.

The largest anomalies in surge levels are often linked to the passage of low pressure systems in the mid-latitudes and with tropical cyclones in the tropics. A proper characterization of the moving speed, track, central pressure drop and size of these atmospheric phenomena is essential to link predicted tropical cyclones to observed surge extremes. Here, we selected the predictor variables from the most recent global reanalysis dataset to ensure a spatially and temporally consistent dataset. While ERA5 can better represent the characteristics of tropical cyclones (position, wind intensity and size) compared to its predecessor product ERA-Interim^28,29,77^, some regions still exhibit large wind biases^30,78^. We expect that these biases do not affect the inter-model comparison because each NN model is independently fit. However, this could affect the spatial patterns of performance. An alternative to reduce this regional bias in future studies could be by including more accurate atmospheric products from local sources such as remote sensing products^10^, even though spatial and temporal data coverage may be limited, or from global forecasting systems^77,79,80^. To improve the representation of tropical cyclones, higher resolution input data could be obtained, for example by applying observed best track data and fitting a parametric wind model^81^, as done for local^25,27^ and global studies^82–84^. To fully harvest the impact of these additional efforts, this should be done in conjunction with a better sampling strategy during training to obtain a more balanced training set^11^.

Our study highlights the complex interplay between hyperparameter optimization, architecture complexity, and the number of predictor variables in model performance. This interplay has not yet been studied in global surge studies using deep learning methods. We show that local model optimization based on deterministic prediction does not necessarily lead to better probabilistic predictions. This means that inter-model comparisons should be carefully interpreted since optimization results can lead to a local optimum in model settings for a few stations rather than a global optimum in model settings for all stations. We found that fitting an ensemble of NN models for each station to provide probabilistic predictions is beneficial to overcome overfitting, unless the ensemble model spread is too large. Nevertheless, the models presented here do not represent a global optimum and further improvements could be made. Because we only carried out the optimization for fifteen stations, optimal settings for other stations or regions could differ from our analysis. Furthermore, using NN instead of hydrodynamic models may lead to several challenges as NN do not help us improve understanding the underlying physical processes of surges, and do not capture long term processes such as sea-level rise or climate variability^32^. In order to face these challenges, future work can make use of physics-informed machine learning to improve the predictive ability and scientific consistency for generalizable NN models^85–87^.

Additional efforts should be closely linked to the intended use of the data to evaluate the best NN architecture,hyperparameters and further developments. For example, including longer term temporal dependence could be done by implementing a stateful LSTM instead of the stateless LSTM implemented in this study. Using our global model settings, the input dimensions in relation to the kernel size of the convolutional layers may be small to evaluate the true performance of these NN as edging effects can occur. Decreasing the edging effects while keeping the high temporal resolution can be a challenge for future research^88,89^. Moreover, this study highlighted the potential of convolutional layers when fed with large enough input for surge predictions, an interesting avenue for further research. For applications in coastal flood risk and extreme sea level analysis, the design of the study should be altered to put an explicit emphasis on extremes and consider total water levels instead of focussing on the surge component only. Our analysis on the influence of the pre-processing steps on sea level return periods (Fig. S3) indicates that errors in the timing of surge residuals can lead to an under or overestimation of extreme sea levels. If compounded with a biased underestimation of extreme surge magnitudes, as observed locally, this could strongly impact reconstructed sea level extremes. A more sophisticated loss function, combining the MAE with other metrics to evaluate extremes, could be implemented to update the layers’ weight and better capture extremes. Similarly, our experiment setup focused on predicting surges based on observed time series, and as such, this method cannot be directly applied for predicting surges at ungauged locations because it requires training data that by definition do not exist. One could circumvent that by applying our approach to modelled data, such as the GTSR dataset^23^.

## Conclusion and outlook

In this study, we have explored deep learning capabilities to predict surges at the global scale. For 738 tide stations, we developed and applied an ensemble approach for four different types of NN to predict hourly surges. We used surge as the predictand variable extracted from observed sea levels of the GESLA-2 dataset and atmospheric variables as predictor variables from the ERA5 climate reanalysis dataset. In order to evaluate the NN model performance at each station, we used the CRPS value and benchmarked the results against a simple probabilistic model based on climatology, i.e. the CRPSS. Next, we explored how increasing the NN design complexity affects model performance by adding hidden layers and enlarging the spatial footprint around each station to extract the predictor variables.

Using the same hyperparameter settings across all stations and a spatial footprint of 1.25 degree to extract predictor variables, the LSTM generally outperforms the other NN types. This is because the probabilistic forecast from the LSTM is in closest agreement with the distribution of the observed values, resulting in the best reliability scores in the CRPS. While the LSTM generally performs best globally when considering a spatial footprint of 1.25 degree, we show that the CNN is capable of improving the most when increasing the spatial footprint or number of hidden layers in the model architecture and outperforms the LSTM. This comes, however, at the expense of increasing computational time, up to more than 15 times longer to run.

Our results show that the NN models are able to capture temporal evolution of surges and outperform large-scale hydrodynamic models. We observe similar performance patterns across all the NN ensemble models, with a performance increasing with latitude and generally high (CRPSS > 40%) in mid-latitudes, which is in line with previous studies. Stations around the equator generally do not outperform the simple probabilistic model based on climatology. Additionally, we show that model performance generally improves with increasing the size of the spatial footprint for the selection of the predictor variables, but that increasing the number of hidden layers does not always lead to a better performance.

Finally, we share the surge input and predicted data and the NN models to invite the coastal community to further build on these initial efforts. We foresee that the NN models developed here could be adapted and tailored for specific coastal applications, for example to provide rapid operational forecast of surge levels, for probabilistic coastal flood hazard assessments, or for future predictions of surges.

## Supplementary Information

Supplementary Information.

## Data Availability

The initial and predicted surge time series at the tide stations analysed, and the NN models at each location in this study are openly available on Zenodo (10.5281/zenodo.5216849). Easy visualisation of the models including the model hyperparameters and features can be accessed in for example with the netron.app website.

## References

[1] Höffken J, Vafeidis AT, MacPherson LR, Dangendorf S (2020). Effects of the temporal variability of storm surges on coastal flooding. Front. Mar. Sci..

[2] Serafin KA, Ruggiero P, Stockdon HF (2017). The relative contribution of waves, tides, and nontidal residuals to extreme total water levels on U.S. West Coast sandy beaches. Geophys. Res. Lett..

[3] Woodworth PL (2019). Forcing factors affecting sea level changes at the coast. Surv. Geophys..

[4] Idier D, Bertin X, Thompson P, Pickering MD (2019). Interactions between mean sea level, tide, surge, waves and flooding: mechanisms and contributions to sea level variations at the coast. Surv. Geophys..

[5] Wu W, Westra S, Leonard M (2017). A basis function approach for exploring the seasonal and spatial features of storm surge events. Geophys. Res. Lett..

[6] Lewis M, Schumann G, Bates P, Horsburgh K (2013). Understanding the variability of an extreme storm tide along a coastline. Estuar. Coast. Shelf Sci..

[7] McInnes KL (2016). Natural hazards in Australia: sea level and coastal extremes. Clim. Change.

[8] Muis S (2020). A high-resolution global dataset of extreme sea levels, tides, and storm surges, including future projections. Front. Mar. Sci..

[9] Cid A, Wahl T, Chambers DP, Muis S (2018). Storm surge reconstruction and return water level estimation in southeast asia for the 20th century. J. Geophys. Res. Ocean..

[10] Tadesse M, Wahl T, Cid A, Lambert E (2020). Data-driven modeling of global storm surges. Front. Mar. Sci..

[11] Bruneau N, Polton J, Williams J, Holt J (2020). Estimation of global coastal sea level extremes using neural networks. Environ. Res. Lett..

[12] Tadesse M, Wahl T (2021). A database of global storm surge reconstruction (GSSR). Sci. Data.

[13] Christie EK (2018). Regional coastal flood risk assessment for a tidally dominant, natural coastal setting: North Norfolk, southern North Sea. Coast. Eng..

[14] Teng J (2017). Flood inundation modelling: A review of methods, recent advances and uncertainty analysis. Environ. Model. Softw..

[15] Santiago-Collazo FL, Bilskie MV, Hagen SC (2019). A comprehensive review of compound inundation models in low-gradient coastal watersheds. Environ. Model. Softw..

[16] Colberg, F. & McInnes, K. L. The impact of future changes in weather patterns on extreme sea levels over southern Australia. *J. Geophys. Res. Ocean.* **117**, (2012).

[17] Nuswantoro R, Diermanse F, Molkenthin F (2016). Probabilistic flood hazard maps for Jakarta derived from a stochastic rain-storm generator. J. Flood Risk Manag..

[18] van den Brink HW, Können GP, Opsteegh JD, van Oldenborgh GJ, Burgers G (2004). Improving 104-year surge level estimates using data of the ECMWF seasonal prediction system. Geophys. Res. Lett..

[19] Cid A, Camus P, Castanedo S, Méndez FJ, Medina R (2017). Global reconstructed daily surge levels from the 20th Century Reanalysis (1871–2010). Glob. Planet. Change.

[20] Chen R, Zhang W, Wang X (2020). Machine learning in tropical cyclone forecast modeling: A review. Atmosphere (Basel)..

[21] Lee TL (2008). Back-propagation neural network for the prediction of the short-term storm surge in Taichung harbour, Taiwan. Eng. Appl. Artif. Intell..

[22] de Oliviera MMF, Ebecken FF, de Oliviera JLF, de Azevedo Santos I (2009). Neural network model to predict a storm surge. J. Appl. Meteorol. Climatol..

[23] Muis S, Verlaan M, Winsemius HC, Aerts JCJH, Ward PJ (2016). A global reanalysis of storm surges and extreme sea levels. Nat. Commun..

[24] Kim S, Matsumi Y, Pan S, Mase H (2016). A real-time forecast model using artificial neural network for after-runner storm surges on the Tottori coast, Japan. Ocean Eng..

[25] Das HS, Jung H, Ebersole B, Wamsley T, Whalin RW (2011). An efficient storm surge forecasting tool for coastal mississippi. Coast. Eng. Proc..

[26] Kim SW, Melby JA, Nadal-Caraballo NC, Ratcliff J (2015). A time-dependent surrogate model for storm surge prediction based on an artificial neural network using high-fidelity synthetic hurricane modeling. Nat. Hazards.

[27] Hashemi MR, Spaulding ML, Shaw A, Farhadi H, Lewis M (2016). An efficient artificial intelligence model for prediction of tropical storm surge. Nat. Hazards.

[28] Hersbach H (2020). The ERA5 global reanalysis. Q. J. R. Meteorol. Soc..

[29] Malakar P, Kesarkar AP, Bhate JN, Singh V, Deshamukhya A (2020). Comparison of reanalysis data sets to comprehend the evolution of tropical cyclones over north indian ocean. Earth Space Sci..

[30] Bian G, Nie G, Qiu X (2021). How well is outer tropical cyclone size represented in the ERA5 reanalysis dataset ?. Atmos. Res..

[31] Shen C (2018). A transdisciplinary review of deep learning research and its relevance for water resources scientists. Water Resour. Res..

[32] Reichstein M (2019). Deep learning and process understanding for data-driven Earth system science. Nature.

[33] Chattopadhyay A, Hassanzadeh P, Pasha S (2020). Predicting clustered weather patterns: A test case for applications of convolutional neural networks to spatio-temporal climate data. Sci. Rep..

[34] Ham YG, Kim JH, Luo JJ (2019). Deep learning for multi-year ENSO forecasts. Nature.

[35] Fang K, Shen C, Kifer D, Yang X (2017). Prolongation of SMAP to spatiotemporally seamless coverage of continental U.S. using a deep learning neural network. Geophys. Res. Lett..

[36] Kratzert F, Klotz D, Brenner C, Schulz K, Herrnegger M (2018). Rainfall: Runoff modelling using Long Short-Term Memory ( LSTM ) networks. Hydrol. Earth Syst. Sci..

[37] Woodworth PL (2017). Towards a global higher-frequency sea level dataset. Geosci. Data J..

[38] Codiga, D. Unified tidal analysis and prediction using the UTide Matlab functions. (2011).

[39] Bevacqua E (2019). Higher probability of compound flooding from precipitation and storm surge in Europe under anthropogenic climate change. Sci. Adv..

[40] Hoitink AJF, Jay DA (2016). Reviews of geophysics tidal river dynamics: implications for deltas. Review Geophys..

[41] Marcos M, Calafat FM, Berihuete Á, Dangendorf S (2015). Long-term variations in global sea level extremes. J. Geophys. Res. Ocean..

[42] Williams J, Irazoqui Apecechea M, Saulter A, Horsburgh KJ (2018). Radiational tides: Their double-counting in storm surge forecasts and contribution to the Highest Astronomical Tide. Ocean Sci..

[43] Hibbert A, Royston SJ, Horsburgh KJ, Leach H, Hisscott A (2015). An empirical approach to improving tidal predictions using recent real-time tide gauge data. J. Oper. Oceanogr..

[44] Brown JM, Bolanos R, Howarth MJ, Souza AJ (2012). Extracting sea level residual in tidally dominated estuarine environments. Ocean Dyn..

[45] Horsburgh KJ, Wilson C (2007). Tide-surge interaction and its role in the distribution of surge residuals in the North Sea. J. Geophys. Res. Ocean..

[46] Haigh ID (2016). Analysis: Spatial and temporal analysis of extreme sea level and storm surge events around the coastline of the UK. Nat. Sci. Data.

[47] Brown JM, Bolaños R, Souza AJ (2014). Process contribution to the time-varying residual circulation in tidally dominated estuarine environments. Estuaries Coasts.

[48] Lyddon C, Brown JM, Leonardi N, Plater AJ (2018). Flood hazard assessment for a hyper-tidal estuary as a function of tide-surge-morphology interaction. Estuaries Coasts.

[49] Rueda A (2017). A global classification of coastal flood hazard climates associated with large-scale oceanographic forcing. Sci. Rep..

[50] Hochreiter S, Schmidhuber J (1997). Long short-term memory. Neural Comput..

[51] Hewamalage H, Bergmeir C, Bandara K (2021). Recurrent neural networks for time series forecasting: current status and future directions. Int. J. Forecast..

[52] Matsugu M, Mori K, Mitari Y, Kaneda Y (2003). Subject independent facial expression recognition with robust face detection using a convolutional neural network. Neural Netw..

[53] Sun W, Su F (2017). A novel companion objective function for regularization of deep convolutional neural networks. Image Vis. Comput..

[54] Xingjian S (2015). Convolutional LSTM network: A machine learning approach for precipitation nowcasting. Adv. Neural Inf. Process. Syst..

[55] Cortes, C., Mohri, M. & Rostamizadeh, A. L2 Regularization for Learning Kernels. *Proc. 25th Conf. Uncertain. Artif. Intell. UAI 2009* 109–116 (2012).

[56] Hertel L, Collado J, Sadowski P, Ott J, Baldi P (2020). Sherpa: Robust hyperparameter optimization for machine learning. SoftwareX.

[57] Wani MA, Bhat FA, Afzal S, Khan AI (2020). Advances in Deep Learning.

[58] Farzad A, Mashayekhi H, Hassanpour H (2019). A comparative performance analysis of different activation functions in LSTM networks for classification. Neural Comput. Appl..

[59] Barbarossa V (2018). FLO1K, global maps of mean, maximum and minimum annual streamflow at 1 km resolution from 1960 through 2015. Sci. Data.

[60] Chollet, F. & others. Keras. Available at: https://github.com/fchollet/keras (2015).

[61] Abadi M (2016). TensorFlow: A System for Large-Scale Machine Learning.

[62] Pedregosa F (2011). Scikit-learn: Machine learning in Python. J. Mach. Learn. Res..

[63] Hersbach H (2000). Decomposition of the continuous ranked probability score for ensemble prediction systems. Weather Forecast..

[64] Trinh BN, Thielen-del Pozo J, Thirel G (2013). The reduction continuous rank probability score for evaluating discharge forecasts from hydrological ensemble prediction systems. Atmos. Sci. Lett..

[65] Pappenberger F (2015). How do I know if my forecasts are better? Using benchmarks in hydrological ensemble prediction. J. Hydrol..

[66] Gilleland, M. *Package ‘verification’*. https://cran.microsoft.com/snapshot/2018-04-09/web/packages/verification/verification.pdf (2015).

[67] Hu Y (2016). A stratified sampling approach for improved sampling from a calibrated ensemble forecast distribution. J. Hydrometeorol..

[68] Janoušek, M. ERA­Interim Daily Climatology. https://confluence.ecmwf.int/download/attachments/24316422/daily_climatology_description.pdf (2011).

[69] Bradley AA, Schwartz SS (2011). Summary verification measures and their interpretation for ensemble forecasts. Mon. Weather Rev..

[70] Resio DT, Westerink JJ (2008). Modeling the physics of storm surges: Physics Today September 2008 Modeling the physics of storm surges. Phys. Today.

[71] Muis S, Haigh ID, Guimarães Nobre G, Aerts JCJH, Ward PJ (2018). Influence of El Niño-southern oscillation on global coastal flooding. Earth’s Futur..

[72] Serafin KA, Ruggiero P, Parker K, Hill DF (2019). What's streamflow got to do with it? A probabilistic simulation of the competing oceanographic and fluvial processes driving extreme along-river water levels. Nat. Hazards Earth Syst. Sci..

[73] Ishii M, Kimoto M (2009). Reevaluation of historical ocean heat content variations with time-varying XBT and MBT depth bias corrections. J. Oceanogr..

[74] Miller L, Douglas BC (2004). Mass and volume contributions to twentieth-century global sea level rise. Nature.

[75] Eilander D (2020). The effect of surge on riverine flood hazard and impact in deltas globally. Environ. Res. Lett..

[76] Ikeuchi H (2017). Compound simulation of fluvial floods and storm surges in a global coupled river-coast flood model: Model development and its application to 2007 Cyclone Sidr in Bangladesh. J. Adv. Model. Earth Syst..

[77] Dullaart JCM, Muis S, Bloemendaal N, Aerts JCJH (2020). Advancing global storm surge modelling using the new ERA5 climate reanalysis. Clim. Dyn..

[78] Belmonte Rivas M, Stoffelen A (2019). Characterizing ERA-Interim and ERA5 surface wind biases using ASCAT. Ocean Sci..

[79] Roberts CD (2018). Climate model configurations of the ECMWF Integrated Forecasting System (ECMWF-IFS cycle 43r1) for HighResMIP. Geosci. Model Dev..

[80] Bloemendaal N, Muis S, Haarsma RJ (2019). Global modeling of tropical cyclone storm surges using high-resolution forecasts. Clim. Dyn..

[81] Lin, N. & Chavas, D. On hurricane parametric wind and applications in storm surge modeling. *J. Geophys. Res. Atmos.* **117**, (2012).

[82] Muis S (2019). Spatiotemporal patterns of extreme sea levels along the western North-Atlantic coasts. Sci. Rep..

[83] Marsooli R, Lin N, Emanuel K, Feng K (2019). Climate change exacerbates hurricane flood hazards along US Atlantic and Gulf Coasts in spatially varying patterns. Nat. Commun..

[84] Bloemendaal N, de Moel H, Muis S, Haigh ID, Aerts JCJH (2020). Estimation of global tropical cyclone wind speed probabilities using the STORM dataset. Sci. Data.

[85] Willard, J., Jia, X., Xu, S., Steinbach, M. & Kumar, V. Integrating physics-based modeling with machine learning: A Survey. *arXiv* **1**, 34 (2020).

[86] Kashinath, K. *et al.* Physics-informed machine learning: Case studies for weather and climate modelling. *Philosophical Transactions of the Royal Society A: Mathematical, Physical and Engineering Sciences* vol. 379 (2021). 10.1098/rsta.2020.0093 33583262

[87] Karpatne A (2017). Theory-guided data science: A new paradigm for scientific discovery from data. IEEE Trans. Knowl. Data Eng..

[88] Innamorati C, Ritschel T, Weyrich T, Mitra NJ (2019). Learning on the edge: Investigating boundary filters in CNNs. Int. J. Comput. Vis..

[89] Nasir V, Sassani F (2021). A review on deep learning in machining and tool monitoring: methods, opportunities, and challenges. Int. J. Adv. Manuf. Technol..

