# Supplementary material for: Exploring deep learning capabilities for surge predictions in coastal areas
Source: Sci Rep. 2021 Aug 26;11:17224. doi: 10.1038/s41598-021-96674-0 (PMC8390491; doi:10.1038/s41598-021-96674-0)
Supplement: Supplementary file 1 — Supplementary Information. [file 41598_2021_96674_MOESM1_ESM.docx]

Supplementary Information to: Exploring deep learning capabilities for surge predictions in coastal areas

Timothy Tiggeloven^1^*, Anaïs Couasnon^1*^, Chiem van Straaten^12^, Sanne Muis^1,3^, Philip J. Ward^1^

^1^Institute for Environmental Studies (IVM), Vrije Universiteit Amsterdam, Amsterdam, The Netherlands

^2^KNMI, Utrechtseweg 297, 3731 GA, De Bilt, Netherlands

^3^Deltares, Delft, The Netherlands

*These authors contributed equally to this work.


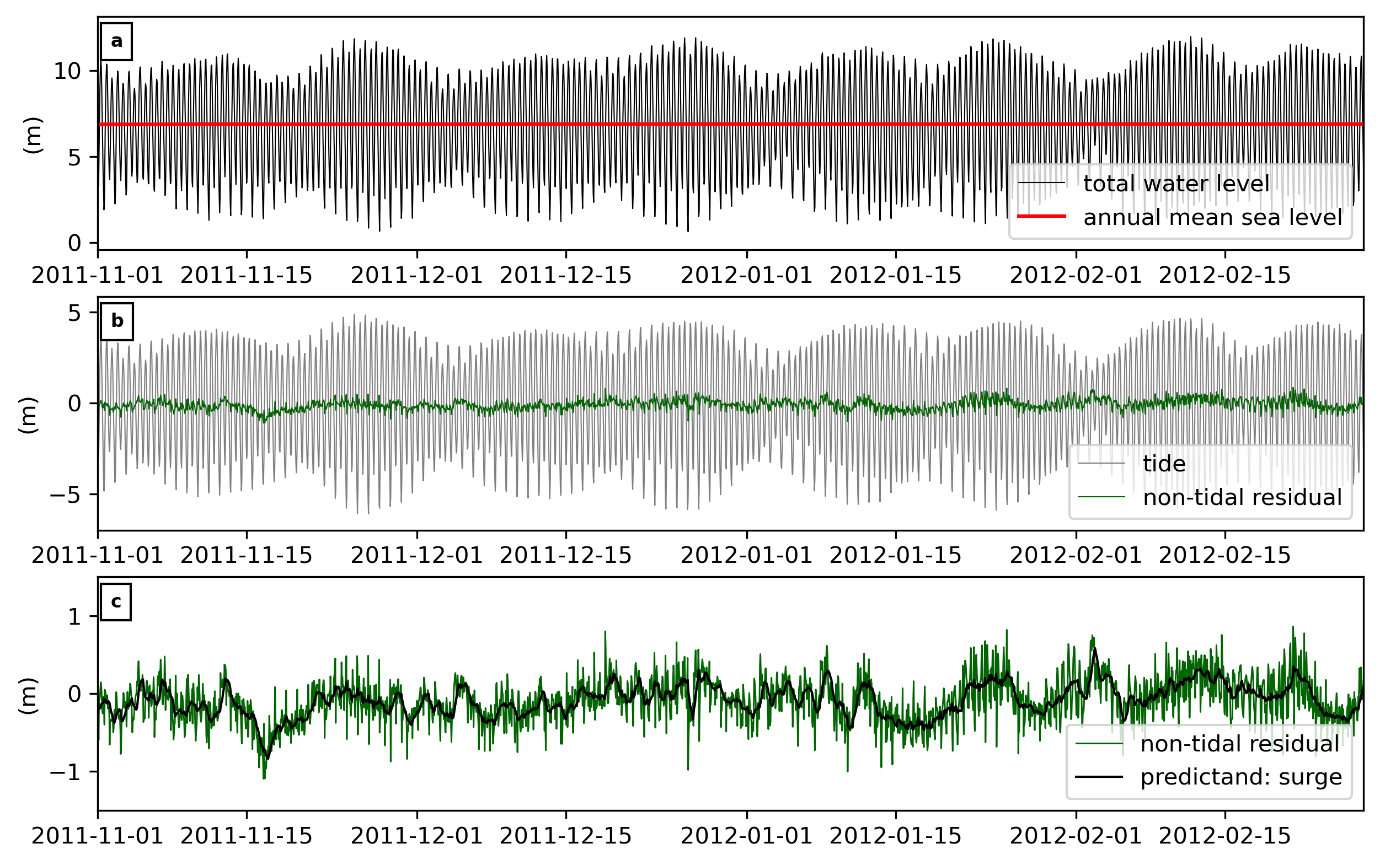


Figure S1: Pre-processing steps applied to the time series of total water level to obtain the surge predictand variable used for this study. a) The annual mean sea level from the total water level from GESLA-2 is subtracted to remove annual mean sea-level variability. b) The detrended total water level is decomposed to obtain the tide and non-tidal residual. c) To limit the impact of harmonic prediction and timing errors, a 12-hour moving average is applied to obtain the surge variable used in this study. The time series shown in from the tidal station Anchorage (location 6 in Figure 1).


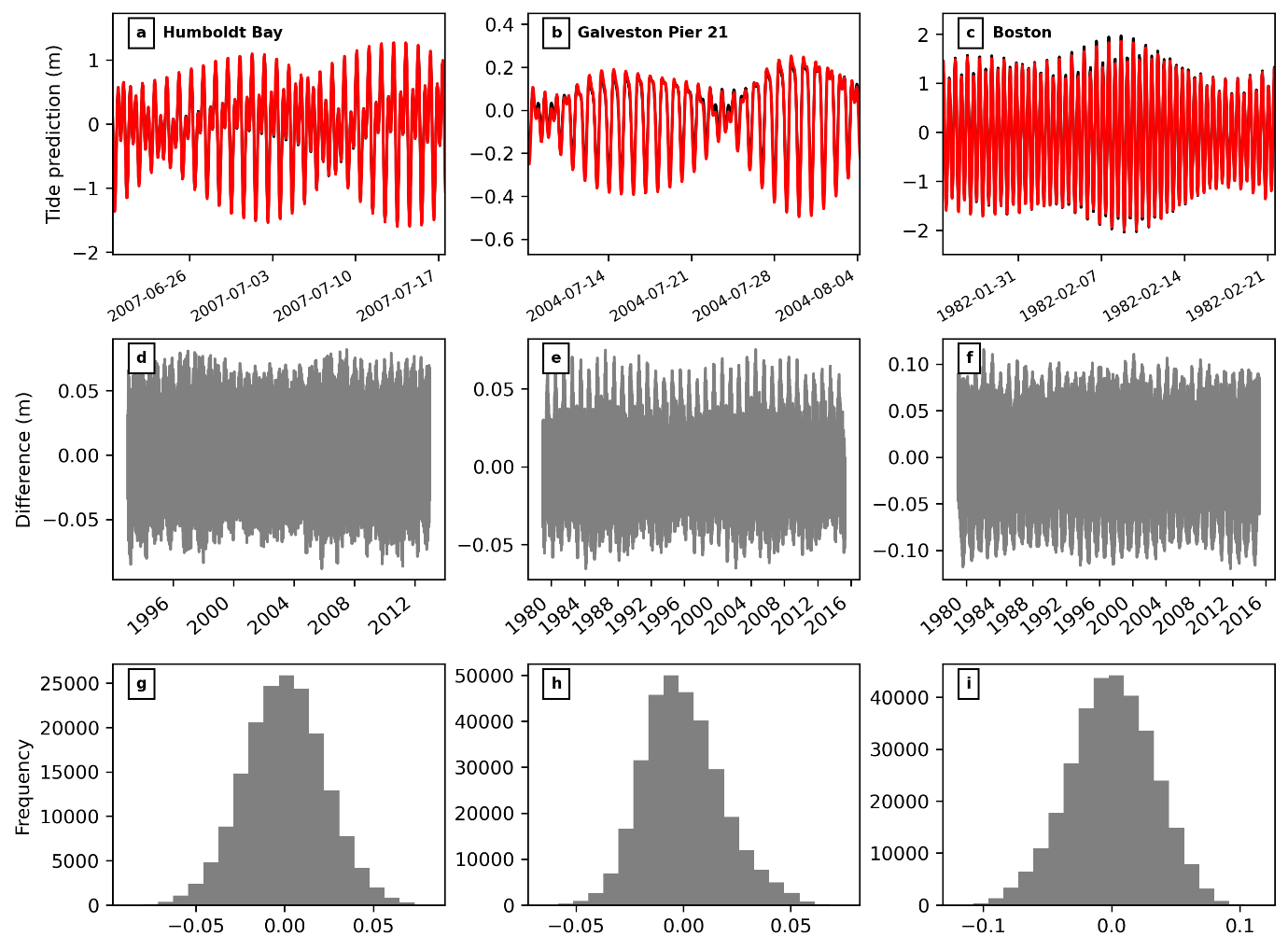


Figure S2: (a-c) Tidal predictions obtained from the UTide package (in black) and NOAA tidal predictions (in red) for a) Humboldt bay (mean tidal range (MTR): 1.49 m), b) Galveston Pier 21 (MTR: 0.31 m) and c) Boston (MTR: 2.89 m). The time span shows the highest differences obtained between both tidal predictions. (d-f) Absolute difference between the two time series. (g-i) Histograms from the absolute differences. NOAA tidal predictions were obtained from <https://tidesandcurrents.noaa.gov/>.


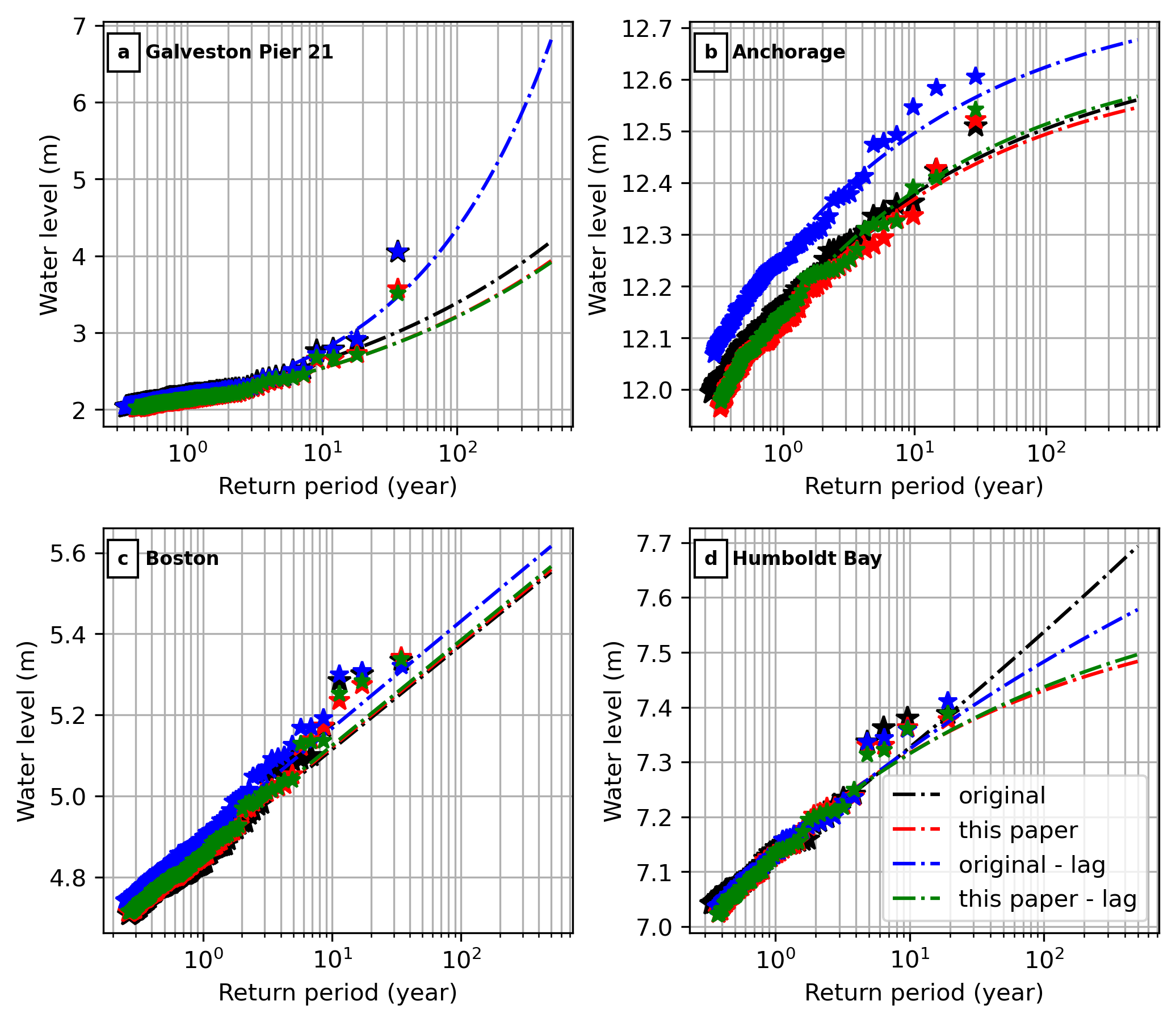


Figure S3: Exceedance frequency and extreme value distribution for the de-trended water level (original, in black), the sum of the tide and non-tidal residual with a lag of 1 hour (original - lag, in blue), the sum of the tide and surge residual derived in this paper (this paper, in red) and the sum of the tide and surge residual with a lag of 1 hour (this paper - lag) for a) Galveston Pier 21, b) Anchorage, c) Boston and d) Humboldt bay.


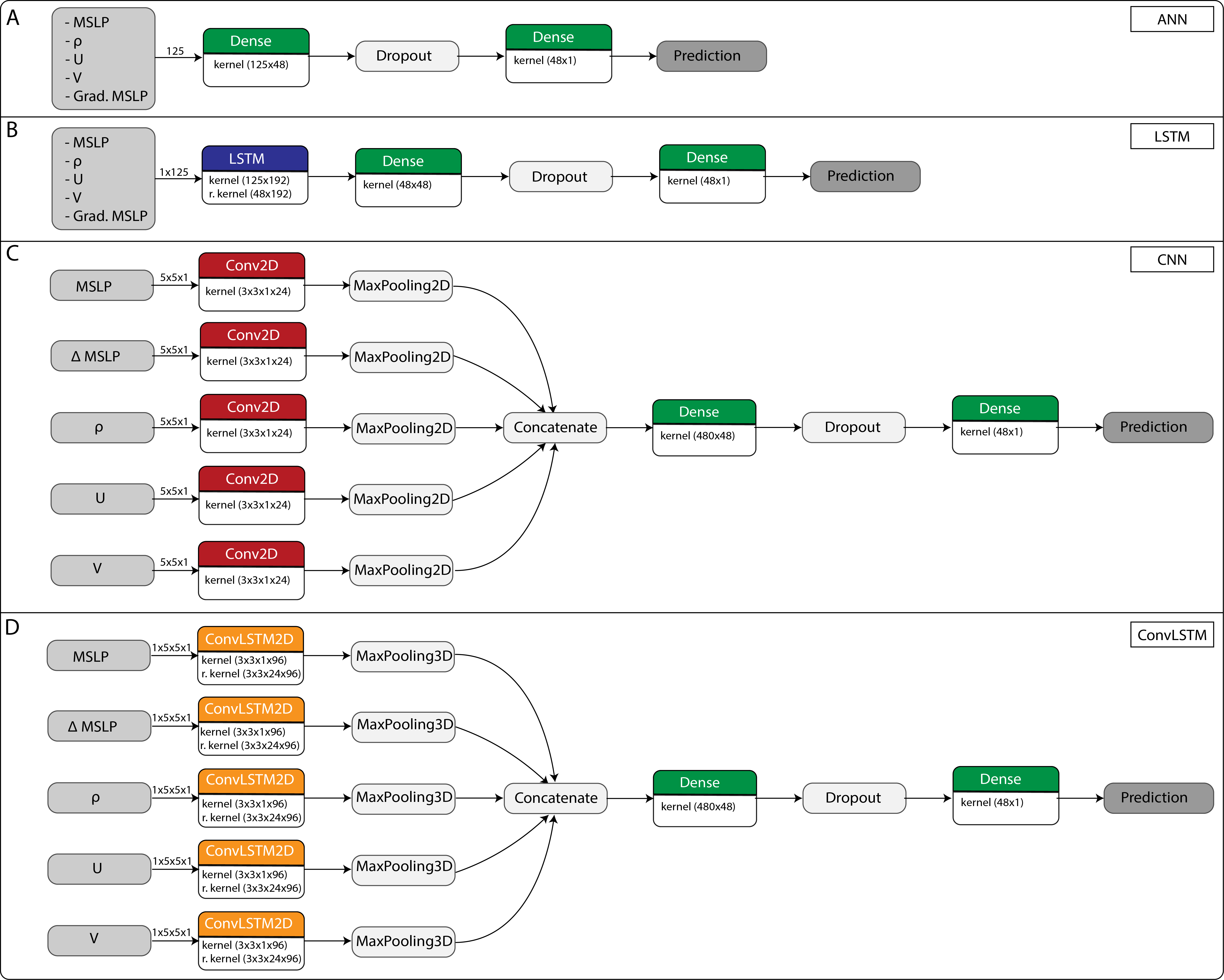


Figure S4: NN architecture for ANN (a), LSTM (b), CNN (c), and ConvLSTM (d) with predictor variable MSLP, gradient of MSLP (Grad. MSLP), wind magnitude (ρ), and U and V. The regular densely connected layers are denoted in green, LSTM layer in blue, convolutional 2D layer for the CNN in red, and the convolutional LSTM 2D layer in orange for the ConvLSTM. For every NN layer the kernel dimensions are shown and for the LSTM and ConvLSTM also the recurrent kernel dimensions are displayed. After the convolutional layers there is a max pooling layer (2D for CNN; 3D for ConvLSTM) to downsample the input and after which input is flattened and concatenated before fed into the densely connected layer. Note that each NN type ends with the same sequence of densely connected layer → dropout → densely connected layer → prediction.


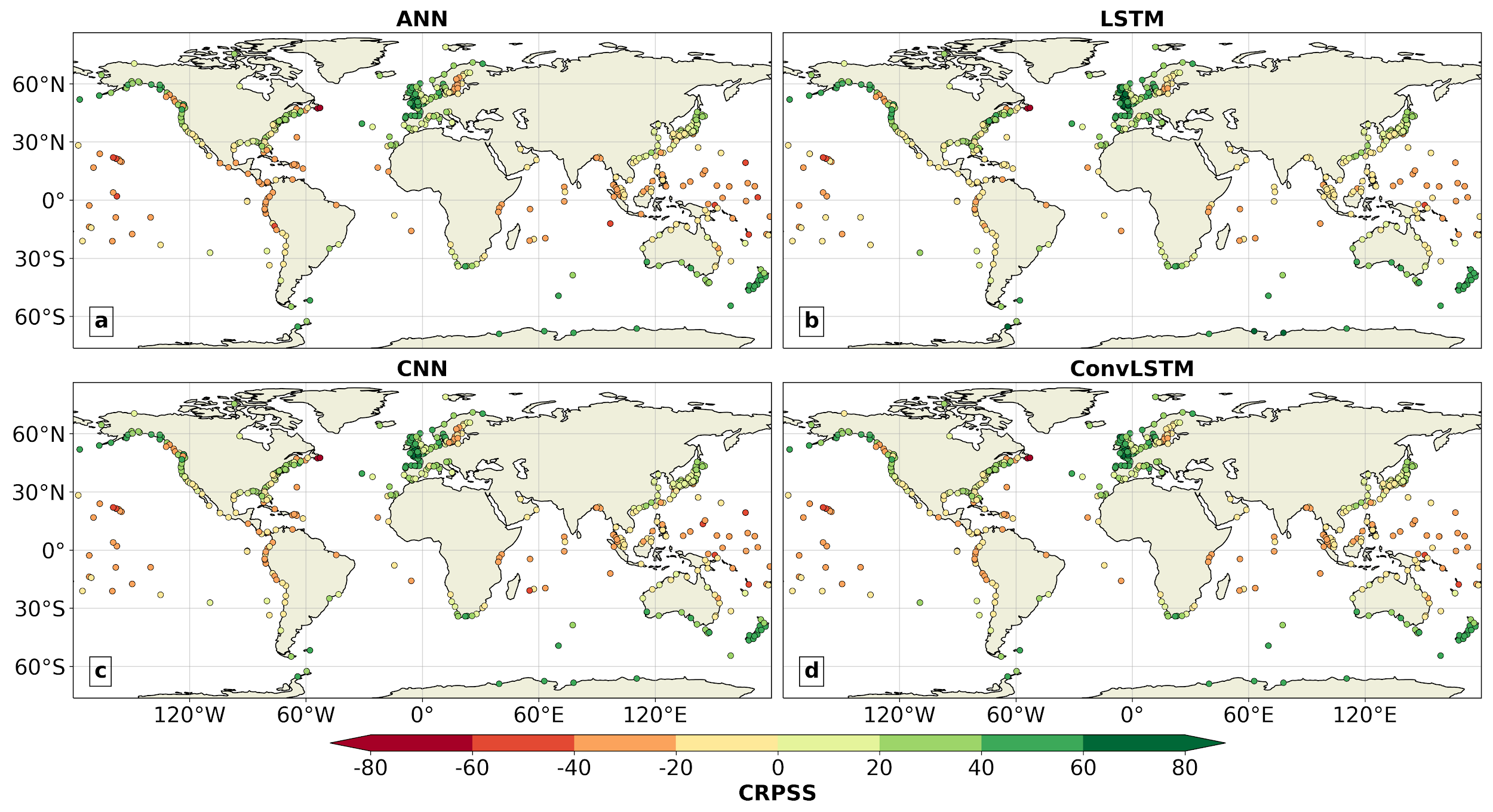


Figure S5: CRPSS of the ensemble run for the four NN.


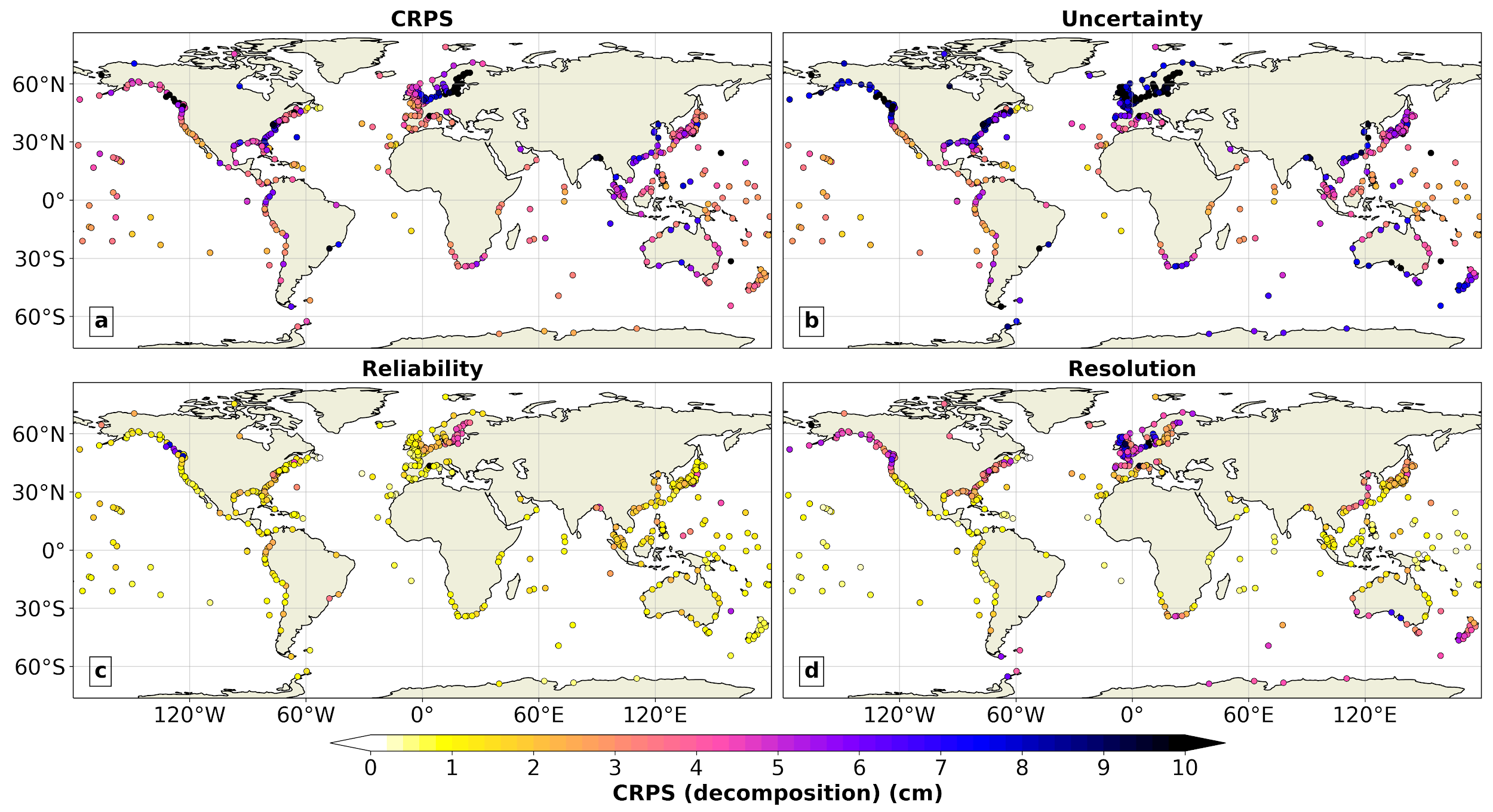


Figure S6: CRPS decomposition for the best NN per tide station. The CRPS value of the climatological reference distribution is expressed as the uncertainty component of the CRPS.


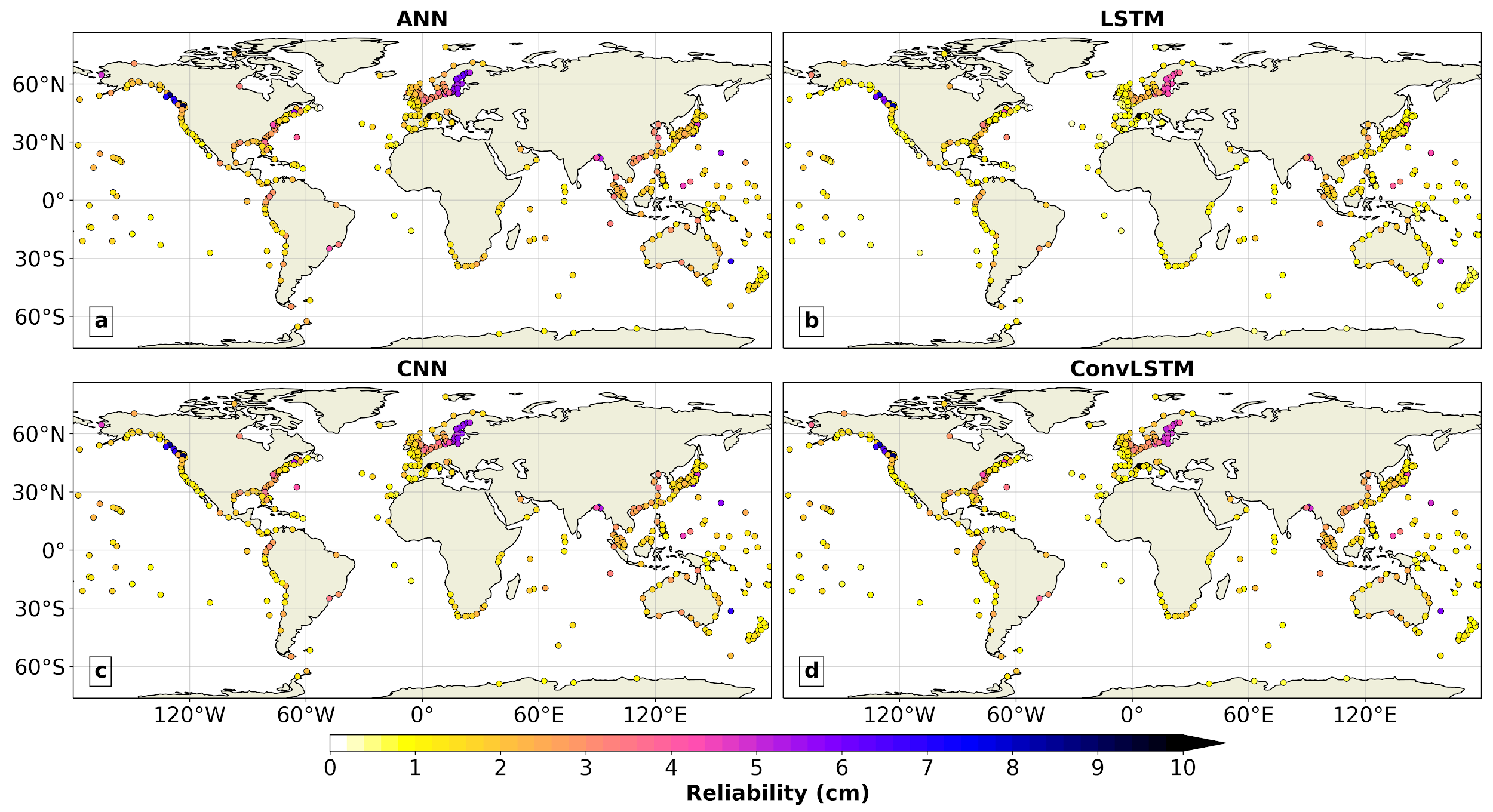


Figure S7: Reliability component of the CRPS for the four NN.


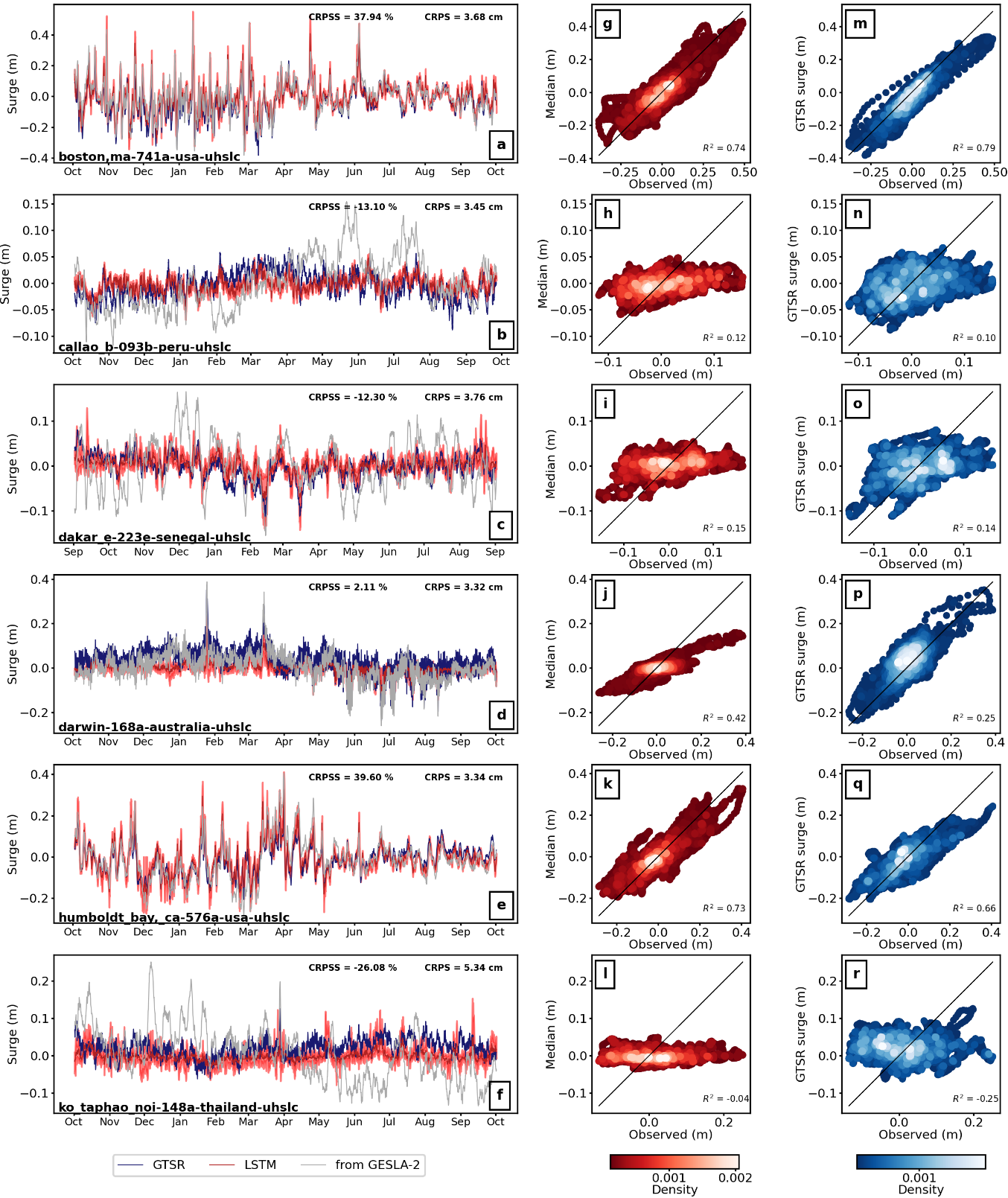


Figure S8: a-f) Hourly surge predictions for the testing year from the LSTM/CNN model, observed and surge from the Global Tide and Surge Model and g-l) scatter plot of the median from the predicted ensemble with the observed surge and m-r) with the GTSM surge.


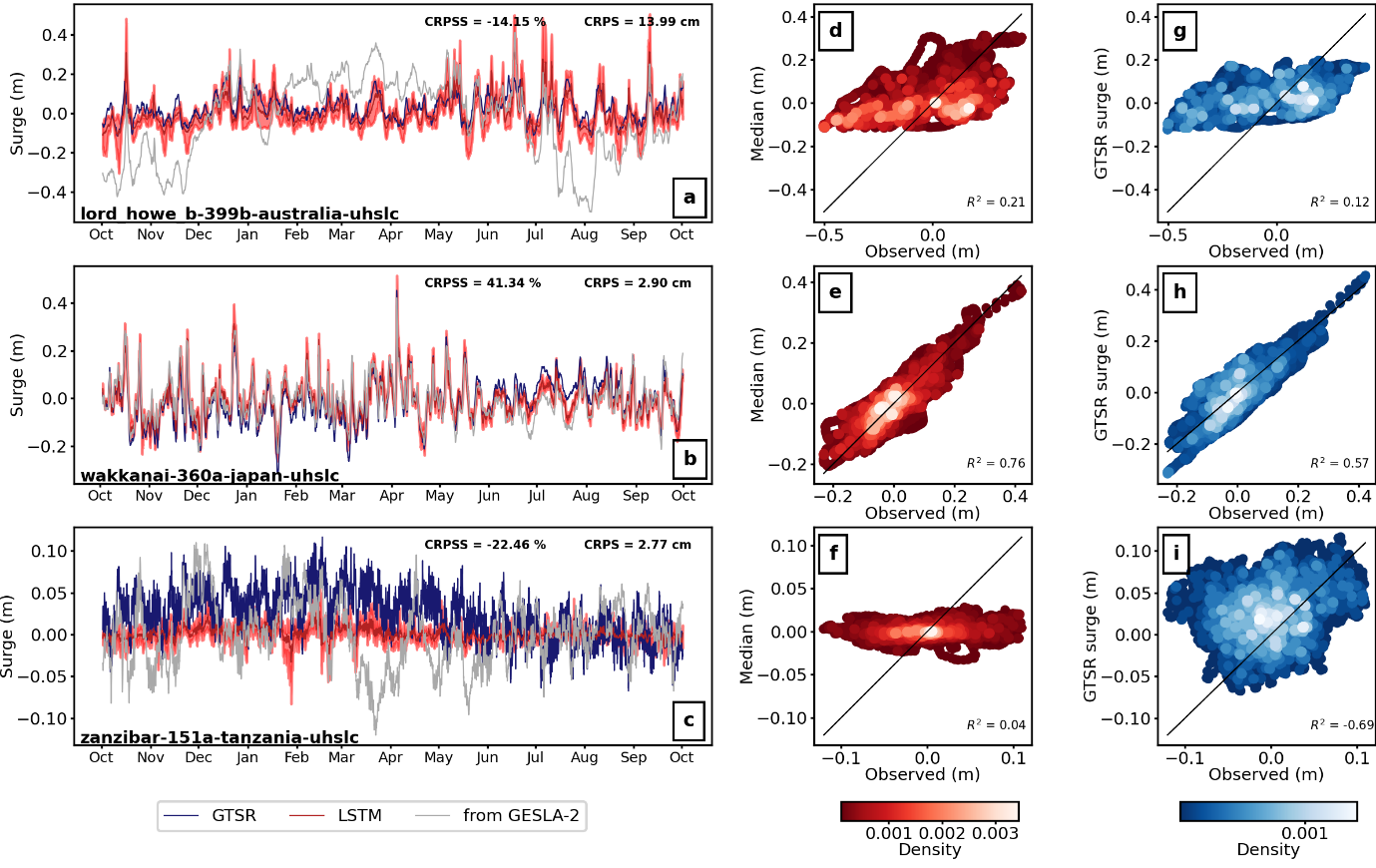


Figure S9: a-c) Hourly surge predictions for the testing year from the LSTM model, observed and surge from the Global Tide and Surge Model and d-f) scatter plot of the median from the predicted ensemble with the observed surge and g-i) with the GTSM surge.


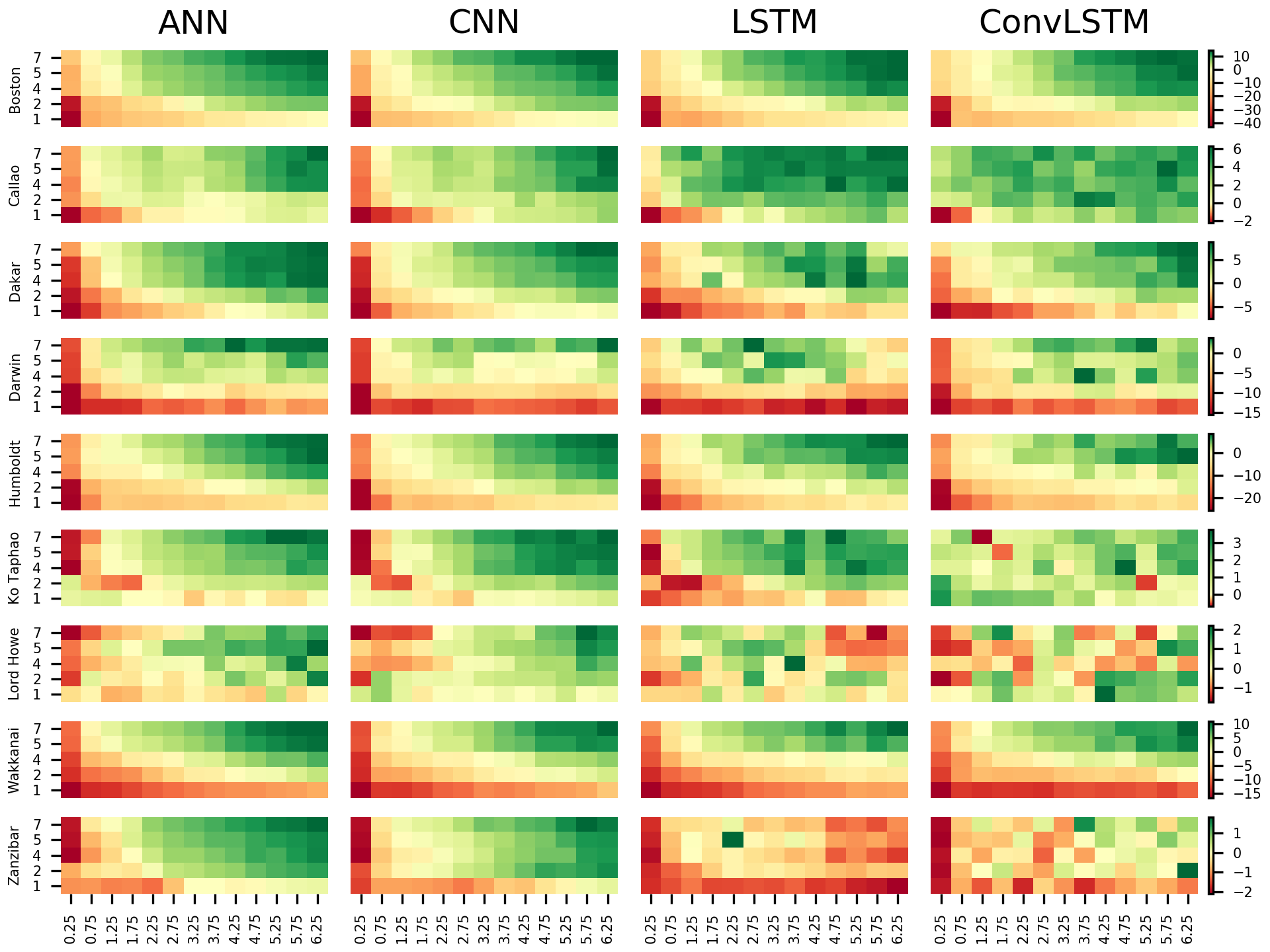
Figure S10: CRPSS value of the NN model ensemble for increasing number predictor variables and spatial footprint size.


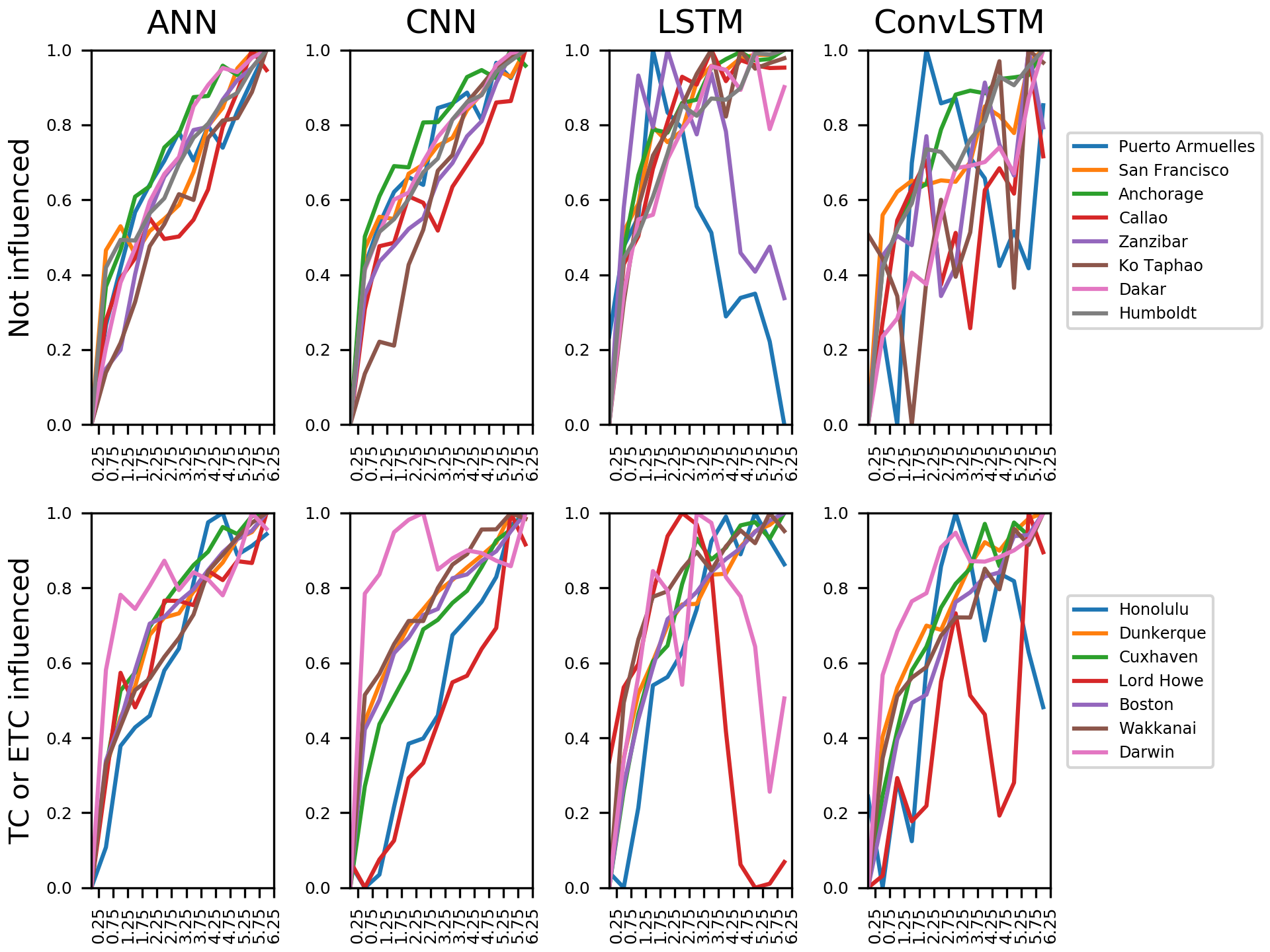


Figure S11: Normalized learning rate of increasing spatial footprint for the four different NN ensembles. The stations have been split into groups of prone to Tropical cyclones (TC) or ExtraTropical Cyclones (ETC) and not influenced by (E)TC based on the IBTrACS dataset, as done in ^1^. Darwin is the only station which was classified as prone to TCs.

1. Bloemendaal, N. *et al.* Generation of a global synthetic tropical cyclone hazard dataset using STORM. *Sci. Data* **7**, 40 (2020).
